# Warming stimulates cellulose decomposition by recruiting phylogenetically diverse but functionally similar microorganisms

**DOI:** 10.1093/ismeco/ycae152

**Published:** 2025-03-03

**Authors:** Yifan Su, Xue Guo, Yamei Gao, Jiajie Feng, Linwei Wu, Jiesi Lei, Suo Liu, Qun Gao, Yufei Zeng, Wei Qin, Zheng Shi, Zhengxiong Liang, Zhencheng Ye, Mengting Yuan, Daliang Ning, Liyou Wu, Jizhong Zhou, Yunfeng Yang

**Affiliations:** Institute of Environment and Ecology, Tsinghua Shenzhen International Graduate School, Tsinghua University, 518071, Shenzhen, China; State Key Joint Laboratory of Environment Simulation and Pollution Control, School of Environment, Tsinghua University, 100084, Beijing, China; State Key Laboratory of Urban and Regional Ecology, Research Center for Eco-Environmental Sciences, Chinese Academy of Sciences, 100085, Beijing, China; College of Life Science and Biotechnology, Heilongjiang Bayi Agricultural University, Daqing, 163710, China; Institute for Environmental Genomics and School of Biological Sciences, University of Oklahoma, Norman, 73019, OK, United States; Institute for Environmental Genomics and School of Biological Sciences, University of Oklahoma, Norman, 73019, OK, United States; Institute of Ecology, Key Laboratory for Earth Surface Processes of the Ministry of Education, College of Urban and Environmental Sciences, Peking University, 100871, Beijing, China; State Key Joint Laboratory of Environment Simulation and Pollution Control, School of Environment, Tsinghua University, 100084, Beijing, China; State Key Joint Laboratory of Environment Simulation and Pollution Control, School of Environment, Tsinghua University, 100084, Beijing, China; Key Laboratory of Water and Sediment Sciences of Ministry of Education and State Key Laboratory of Water Environment Simulation, School of Environment, Beijing Normal University, 100875, Beijing, China; State Key Joint Laboratory of Environment Simulation and Pollution Control, School of Environment, Tsinghua University, 100084, Beijing, China; Institute for Environmental Genomics and School of Biological Sciences, University of Oklahoma, Norman, 73019, OK, United States; Institute for Environmental Genomics and School of Biological Sciences, University of Oklahoma, Norman, 73019, OK, United States; State Key Joint Laboratory of Environment Simulation and Pollution Control, School of Environment, Tsinghua University, 100084, Beijing, China; State Key Joint Laboratory of Environment Simulation and Pollution Control, School of Environment, Tsinghua University, 100084, Beijing, China; Institute for Environmental Genomics and School of Biological Sciences, University of Oklahoma, Norman, 73019, OK, United States; Department of Environmental Science, Policy, and Management, University of California, Berkeley, 94720, CA, United States; Institute for Environmental Genomics and School of Biological Sciences, University of Oklahoma, Norman, 73019, OK, United States; Institute for Environmental Genomics and School of Biological Sciences, University of Oklahoma, Norman, 73019, OK, United States; Institute for Environmental Genomics and School of Biological Sciences, University of Oklahoma, Norman, 73019, OK, United States; School of Civil Engineering and Environmental Sciences, University of Oklahoma, Norman, 73019, OK, United States; Earth and Environmental Sciences, Lawrence Berkeley National Laboratory, Berkeley, 94720, CA, United States; Institute of Environment and Ecology, Tsinghua Shenzhen International Graduate School, Tsinghua University, 518071, Shenzhen, China; State Key Joint Laboratory of Environment Simulation and Pollution Control, School of Environment, Tsinghua University, 100084, Beijing, China

**Keywords:** cellulose decomposition, litterbags, soil respiration, microbial functional traits, warming effects, temperate grasslands

## Abstract

Cellulose is the most abundant component of plant litter, which is critical for terrestrial carbon cycling. Nonetheless, it remains unknown how global warming affects cellulose-decomposing microorganisms. Here, we carried out a 3-year litterbag experiment to examine cellulose decomposition undergoing +3°C warming in a tallgrass prairie. Most cellulose-associated bacteria and fungi in litterbags were also detected in bulk soil, and bacteria in litterbags had higher community-level *rrn* copy numbers, larger genome sizes, and higher genome guanine-cytosine (GC) contents than those in bulk soil, implying higher growth rates. Warming stimulated soil respiration by 32.3% and accelerated mass loss of cellulose, concurring with the increase in relative abundances of most functional genes associated with carbon decomposition in litterbags. Incorporating cellulose-decomposing genes into an ecosystem model reduced model parameter uncertainty and showed that warming stimulated microbial biomass, activity, and soil carbon decomposition. Collectively, our study supports a trait-centric view since cellulose-decomposing genes or genomic traits are amenable for ecosystem modeling. By characterizing the phylogenetically diverse yet functionally similar cellulose-associated microorganisms and their responses to warming, we take a step toward more precise predictions of soil carbon dynamics under future climate scenarios.

## Graphical Abstract

**Graphical Abstract:**
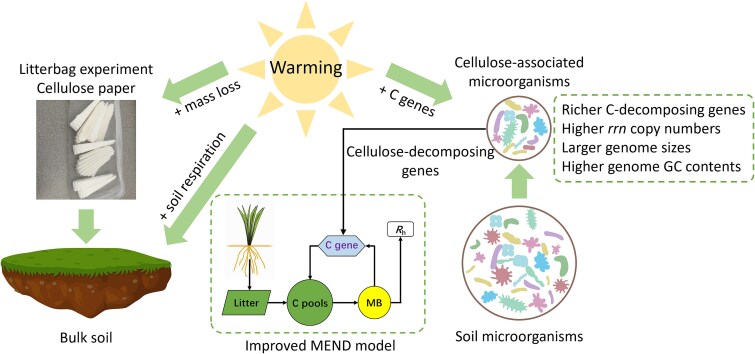

## Introduction

Microbial decomposition of organic matter is crucial for the cycling of carbon and other nutrients in terrestrial ecosystems [1, 2]. This process fosters the allocation of plant litter carbon to the soil carbon pool, which is essential for maintaining carbon balance [3]. Since the enzyme activity [4], biomass [5], diversity [6], and composition [7] of soil microbial communities are intimately affected by temperature, global warming has the potential to accelerate microbial decomposition rates, profoundly affecting soil carbon flux and stock [8]. Therefore, understanding microbial communities and their carbon-decomposing capacities under warming could improve our prediction about the direction and magnitude of future climate changes.

Plant litter is the primary source of aboveground carbon input to soils in a tallgrass prairie, where 19% of aboveground plant litter carbon loss becomes soil organic carbon, while the rest is mineralized to CO_2_ [9]. Cellulose typically accounts for 35%–50% of plant dry weight [10], serving as the main substrate during grass litter decomposition [11]. Although cellulose is more labile than recalcitrant litter compounds, such as lignin and alkyl carbon, it is insoluble and requires hydrolyzation into small oligosaccharides by extracellular enzymes produced by microorganisms, such as endoglucanases and cellobiohydrolases [12].

Warming could accelerate litter decomposition if a warming-induced decrease in soil moisture does not limit microbial enzymatic activity [13, 14], which is contingent upon the compositions and functions of microbial communities that regulate litter decomposition [15, 16]. As previously observed, warming increased the proportion of gram-positive bacteria in fresh litter, but decreased it in the lower-quality pre-incubated litter [17]. Warming favored ectomycorrhizal fungi but decreased the relative abundance of saprotrophs and plant pathogens, correlating with changes in different kinds of extracellular enzyme activities [18]. In macrophyte litter, warming shifted the functional composition of bacterial communities, decreasing the main chemoheterotrophic metabolic potential and increasing methylotrophic metabolism [19]. However, little is understood about the functional traits of plant litter decomposers in soil. For example, genome-wide functional traits, such as ribosomal ribonucleic acid (RNA) gene operon (*rrn*) copy number, genome size, and guanine-cytosine (GC) content, have never been examined for plant litter decomposers. These traits are closely related to microbial growth rate and carbon use efficiency [20], and thus are crucial in predicting microbial performance responses to future climate changes.

The effects of warming on litter decomposition are influenced by the species and quality of the litter [21, 22]. Therefore, we focus on the decomposition of cellulose to eliminate confounding factors, such as litter species and quality. Here, we carried out a litterbag experiment that used cellulose paper as the substrate. To ensure the reliability of our results, we repeated the experiment for three consecutive years by burying litterbags in the soils of a temperate, humid tallgrass prairie in each year. Since the cellulose-decomposing rate was fast in this type of ecosystem [23], most of the cellulose paper was decomposed after 6 months in our pre-experiment. Here, we aimed to investigate the taxonomic composition and functional traits of soil cellulose decomposers, as well as the effects of in situ experimental warming on these parameters. We hypothesized that litterbags would recruit taxonomically diverse but functionally similar microbial taxa in cellulose decomposition. We further hypothesized that warming would alter the taxonomic composition and increase the carbon decomposition potential of these microbial communities, since carbon decomposition potential is a key microbial functional trait that reflects the ability of microorganisms to accelerate the breakdown of organic carbon compounds in response to warming.

## Materials and methods

### Site description and field measurements

We conducted this study at the Kessler Atmospheric and Ecological Field Station of the US Great Plains in McClain County, Oklahoma, USA (34^o^ 59′N, 97^o^ 31′W). As described previously [7], this station is located in an old-field tall-grass prairie dominated by C3 grass (*Bromus arvensis* and *Bromus racemosus*), C3 forbs (*Solanum carolinense*, *Croton glandulosus*, and *Euphorbia dentata*), and C4 grasses (*Sorghum halepense* and *Tridens flavus*). The mean annual air temperature was 16.3°C, with monthly air temperature ranging from 3.3°C in January to 28.1°C in July, and the mean annual precipitation was 914 mm based on the Oklahoma Climatological Survey data from 1948 to 1999. The field site experiment was established in July 2009, using a blocked split-plot design with warming as a primary factor. Four plots with the size of 2.5 × 1.75 m were warmed by real infrared heaters (Kalglo Electronics, Bath, PA, USA) suspended 1.5 m above the ground, while the other four control plots of the same size were exposed to dummy heaters that simulated a shading effect. Soil temperature, moisture, ecosystem carbon fluxes, above-ground plant biomass, and richness were measured in the field as described previously [2, 7].

### Sample collection and soil geochemistry

In each year from 2016 to 2018, three litterbags containing 5 g of sterile cellulose paper were buried at a soil depth of 5–10 cm in each plot in March. By September of the same year, most of the cellulose paper in litterbags had decomposed. All litterbags were then retrieved from the field. For each plot, one litterbag was stored at −80°C for deoxyribonucleic acid (DNA) extraction from the remaining cellulose paper and the soil it carried, while the other two litterbags were gently washed with water and then dried to calculate the mass loss. To obtain soil samples, three soil cores (2.5 × 15 cm deep) were randomly collected for each plot and mixed to make one soil sample. As a result, 12 (4 samples per year × 3 years) warmed litterbag (WL) samples, 12 control litterbag (CL) samples, 12 warmed bulk soil samples, and 12 control bulk soil samples were obtained (Supplementary Fig. 1). However, we did not obtain credible mass loss data in 2018 because the litterbag samples were not properly processed. After removing visible roots (> 0.25 cm) and stones, organic C, total N, nitrate (NO_3_^−^-N), ammonia (NH_4_^+^-N), and pH were measured for bulk soil samples as previously described [7].

### Deoxyribonucleic acid extraction, amplicon sequencing, and GeoChip hybridization

Microbial DNA was extracted for each sample by freeze-grinding and SDS-based cell lysis [24], and purified with the PowerSoil DNA Isolation Kit (MoBio Laboratories, Carlsbad, CA, USA). The quality of extracted DNA was assessed by the absorbance ratios of O.D. 260/230 nm and O.D. 260/280 nm with an ND-1000 Spectrophotometer (NanoDrop Technologies Inc., Wilmington, DE, USA). The DNA concentration was quantified using a FLUOstar Optima fluorescence plant reader (BMG Labtech, Jena, Germany).

The V4 hypervariable region of bacterial 16S ribosomal RNA (rRNA) gene was polymerase chain reaction (PCR)-amplified with the universal primer pair 515F (5’-GTGCCAGCMGCCGCGGTAA-3′) and 806R (5′-GGACTACHVGGGTWTCTAAT-3′). The fungal internal transcribe sequences (ITS) between 5.8S and 28S rRNA genes was amplified with the primer pair gITS7F (5′-GTGARTCATCGARTCTTTG-3′) and ITS4R (5′-TCCTCCGCTTATTGATATGC-3′). A two-step PCR approach was used to prepare libraries [7, 25]. The PCR products were sequenced using the 2 × 250 bp kit on a MiSeq platform (Illumina, San Diego, CA, USA). The reads collected by the MiSeq in fastq format were processed in USEARCH v.11.0 [26]. In brief, after demultiplexing, paired-end reads with a maximum of 10 mismatches and a minimum alignment identity of 80% were merged to create consensus sequences. Subsequently, primer and low-quality sequences with an expected error > 1 were trimmed. After removing singletons, sequences were denoised into zero-radius operational taxonomic units (zOTUs) by UNOISE3 [27]. Sequences were randomly resampled to the same depth of 30 236 sequences for the 16S rRNA and 10 391 sequences for the ITS for each sample. Representative sequences for each zOTU were taxonomically classified using QIIME2 feature classifier with the SILVA reference database for 16S rRNA genes and the UNITE-released ITS reference database for ITS genes [28].

We employed the GeoChip 5.0 M microarray to examine microbial functional genes as previously described [29–31]. In brief, purified DNA from each sample was labeled with fluorescent dye Cy-3 dUTP and then hybridized with the GeoChip. The resulting GeoChip arrays were scanned as Multi-TIFF images with a NimbleGen MS200 Microarray Scanner (Roche NimbleGen Inc., Madison, WI, USA). Raw signals extracted from scanned images were submitted to the Microarray Data Manager on our website (http://ieg.ou.edu/microarray/) for cleaning and normalizing. To remove unrepresentative data, we assigned probes detected in <3 out of 12 samples for WL, CL, warmed soil (WS), and control soil (CS) samples to undetectable. We then logarithmically transformed the remaining data and scaled the sum of the signal intensity for each sample with the maximum sum value.

### Estimation of prokaryotic rrn copy number, genome size, and GC content

The *rrn* copy number, genome size, and GC content for prokaryotic zOTUs were estimated as previously described [32]. We downloaded 32 701 prokaryotic “Complete Genome” assemblies deposited in Genbank from NCBI on 23 November 2022 (ftp://ftp.ncbi.nlm.nih.gov/genomes/GENOME_REPORTS/prokaryotes.txt) and constructed a local database. We used BLAST to identify complete genomes that have the best match (with the highest bit score) at the 16S rRNA locus with our representative sequences. In total, 21.56% of sequences were matched with a chromosome or plasmid at 94.5% identity. We estimated the number of copies of the 16S rRNA gene for each best-matched genome by counting the number of iterations in the genome that contained the match to our sequence. For zOTUs with multiple best-matched genomes, we determined the *rrn* copy number for each zOTU as the average copy number of all the best-matched genomes. We then calculated the community-level *rrn* copy number for each sample by dividing the sum of the sequence abundance of zOTUs by the sum of the copy number-adjusted abundance of zOTUs [33, 34]. Additionally, we estimated the genome size and genome GC content for each zOTU based on best-matched genomes. The abundance-weighted genome size and abundance-weighted genome GC content were calculated as the average weighted by relative abundance [32].

### Network analyses

We constructed molecular ecological networks (MENs) by a random matrix theory (RMT)-based approach using the Functional Molecular Ecological Network Analysis Pipeline (http://ieg4.rccc.ou.edu/mena) as previously described [35–37]. This co-occurrence network was widely used to infer putative microbial species interaction [38]. Specifically, we constructed four bacterial and four fungal networks using taxonomic zOTU data for WL, CL, WS, and CS samples, respectively. Only zOTUs detected in at least 6 out of 12 samples were included for Spearman correlation calculation using log-transformed relative abundances. We generated sub-networks for each sample from the global networks as previously described [39, 40], and calculated network topological properties for sub-networks including average degree, average clustering coefficient (avgCC), average path distance (geodesic distance), number of modules, modularity, and density.

### Microbial-ENzyme Decomposition model simulations

The Microbial-ENzyme Decomposition (MEND) model exhibits a reasonable fit to soil carbon fluxes by incorporating pertinent microbial and enzymatic physiology [41]. The model’s structure framework has been described previously [31, 42]. In brief, the soil organic matter (SOM) pool comprises particulate organic matter decomposed by oxidative enzymes (POM_1_), POM decomposed by hydrolytic enzymes (POM_2_), and mineral-associated organic matter (MOM) decomposed by generic enzyme group (EM). In the MEND model, the decomposition of POM and MOM, as well as the uptake of dissolved organic matter (DOC) follow the Michaelis–Menten equation. The temperature response functions of kinetics parameters are described by the Q_10_ method in this study. More details regarding variables, governing equations, component fluxes, and model parameters can be found in published papers [31, 43].

We cleaned the daily soil temperature and moisture data, addressing missing values by fitting them with data from a nearby weather station. We obtained the daily gross primary production (GPP) values from the 8-day MODIS GPP (MOD17A2H) using Google Earth Engine and employed them as litter fall input for the model simulations [44]. Eleven parameters related to carbon fluxes and enzymes were selected for optimization and other parameters were set to default values. The objective functions measured the goodness-of-fits of model simulations against experimental observations on heterotrophic respiration (*R*_h_) (*J*_1_), microbial biomass carbon (MBC) (*J*_2_), and abundances of genes associated with cellulose decomposition (*J*_3_) in this study. *J*_1_ and *J*_2_ were calculated as the coefficient of determination, and *J*_3_ was determined as the correlation coefficient. The optimal parameter values were derived by minimizing the total objective function value with the modified Shuffled Complex Evolution algorithm [42]. In addition, the parameter uncertainty of the MEND model was quantified by the Critical Objective Function Index (COFI) method [42].

### Statistical analyses

We examined the difference in environmental factors, α-diversity, relative abundance, genome traits, and network properties among WL, CL, WS, and CS samples using a linear mixed model (LMM) with block and year as random effects [45]. We also used LMM to correlate taxonomic richness and functional richness calculated from GeoChip data. The coefficient (*r*^2^) was calculated using Nakagawa and Schielzeth’s method (conditional *r*^2^) [46]. The significance of each LMM was determined using Wald type II χ^2^ tests conducted with the 'car' R package [47]. We used principal coordinates analyses based on Bray–Curtis distance and Sørenson distance to illustrate the difference in compositions, and tested them using three non-parametric multivariate statistical tests including permutational multivariate analysis of variance (Adonis), analysis of similarity (Anosim), and multiple response permutation procedure (MRPP). We assessed within-group dispersion of community and functional gene compositions by the average Bray–Curtis distance to centroid using “betadisper” function of the package 'vegan' in R [48], and compared dispersion among groups using ANOVA and Tukey test. We performed differential abundance analysis using EdgeR’s quasi-likelihood negative binomial generalized log-linear model and ALDEx2 [49, 50]. We determined overlapped zOTUs identified by both EdgeR’s quasi-likelihood negative binomial generalized log-linear model and ALDEx2 as keystones in the litterbags compared to bulk soils and illustrated the phylogenetic tree of bacterial keystones using iTOL v.6.7 [51]. We calculated the response ratio (RR) to compare the abundance of microbial genes involved in carbon decomposition between litterbags and bulk soils and examine the warming effects on these genes in litterbags and bulk soils [52]. We used the partial Mantel test to examine the linkage between microbial community compositions and environmental factors. We used multiple regression on matrices (MRM) to estimate the importance of abiotic and biotic factors to the variation in microbial compositions, and topological properties of sub-networks were regarded as biotic factors [40]. We used normalized stochasticity ratios based on taxonomic metrics (tNST) to estimate the relative significance of stochasticity in shaping microbial community with 50% as the boundary point [53]. Specifically, the tNST was calculated by constructing a null model that randomized species composition. The observed community dissimilarity was then compared to this null distribution, and the normalized stochasticity ratio was calculated as the proportion of pairwise comparisons where observed dissimilarity exceeded that of the null model. A value greater than 50% reflects the dominance of stochastic processes, while a value <50% reflects the dominance of deterministic processes. We assessed the relationship between taxonomic and functional composition dissimilarities by Mantel tests based on Bray–Curtis distance [54].

## Results

### Taxonomic compositions of bacterial and fungal communities

**Figure 1:**
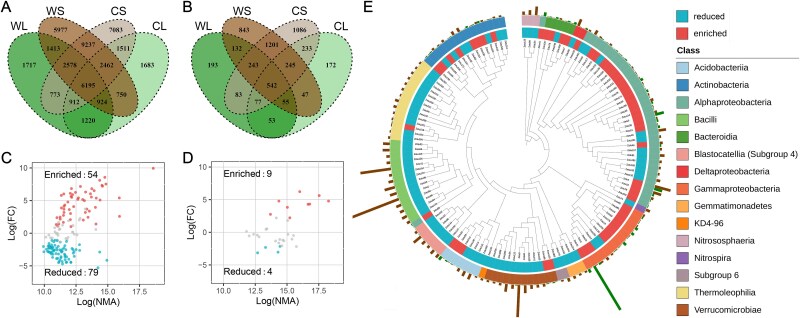
Enriched taxa and reduced taxa in litterbags. Venn diagrams showing the shared and unique (A) bacterial and (B) fungal zOTUs in different samples. The volcano plot shows the enrichment and reduction of (C) bacterial and (D) fungal zOTUs in litterbags compared to bulk soil. These zOTUs had relative abundance ≥0.1% in at least 6 samples and occurred in >12 samples. NMA, normalized mean abundance; FC, fold change. (E) Phylogenetic tree and taxonomic composition of enriched and reduced bacterial taxa. The columns in the outer ring represent the relative abundance of zOTUs.

The bacterial and fungal richness and Shannon's diversity index were lower in litterbags than in bulk soil (*P* < .001 by LMM; Supplementary Fig. 2A and B). Most bacteria (79.1%) and fungi (79.9%) detected in litterbags were shared with bulk soil (Fig. 1A and B), suggesting that soil was the primary source of microorganisms in litterbags. The community compositions were distinct between the litterbags and bulk soils (principal coordinates analyses, Supplementary Fig. 3), whose differences were statistically significant, as examined by three complementary non-parametric multivariate statistical analyses (*P* < .001 by Adonis, Anosim, and MRPP; Supplementary Table 1). In bulk soil, Proteobacteria (also refer as Pseudomonadota, 30.0% of total abundance) and Actinobacteria (also refer as Actinomycetota, 20.1% of total abundance) were the most abundant bacterial phyla (Supplementary Fig. 4A). In litterbags, the total abundance of Proteobacteria rose to 61.4% (F = 175.850, *P* < .001 by LMM), followed by Bacteroidetes (also refer as Bacteroidota, 12.7% of total abundance), but that of Actinobacteria decreased to 8.0% (Supplementary Fig. 4A; F = 93.810, *P* < .001 by LMM). Ascomycota and Basidiomycota were the dominant fungal phyla in both bulk soil and litterbags, with the total abundance of Ascomycota increasing from 56.0% in bulk soil to 73.9% in litterbags (F = 9.734, *P* < .010 by LMM; Supplementary Fig. 4B). Furthermore, the taxonomic composition of microbial communities in litterbags showed higher dispersion than that in bulk soil (*P* < .050 by ANOVA; Table 1), suggesting that they were more heterogeneous.

**Table 1:** The within-group dispersion of microbial communities.

Method	Average distance to the centroid		Significance
	Litterbag	Soil	
Bacterial communities			
Bray–Curtis	0.504	0.384	**<0.001** ^**a**^
Sørenson	0.480	0.411	**<0.001**
Fungal communities			
Bray–Curtis	0.609	0.573	**0.021**
Sørenson	0.527	0.482	**<0.001**
Functional genes			
Bray–Curtis	0.196	0.078	**<0.001**
Sørenson	0.151	0.059	**<0.001**

a Significant differences (*P* < .050) are shown in bold.

Out of 163 abundant bacterial zOTUs (defined as having a total abundance of ≥0.1% in at least 6 samples and occurring in >12 samples), 54 were enriched in litterbags and 79 were reduced in litterbags (*P* < .050 by EdgeR and ALDEx2; Fig. 1C). Out of 32 abundant fungal zOTUs, 9 were enriched and 4 were reduced in litterbags (Fig. 1D). Those enriched bacterial zOTUs mainly belonged to the phyla Proteobacteria (66.7%) and Actinobacteria (16.7%), as well as the class Bacteroidia of the phylum Bacteroidetes (Fig. 1E). Those reduced bacterial zOTUs mainly belonged to the class Thermoleophilia of the phylum Actinobacteria, the order Bacillales of the phylum Firmicutes (also refer as Bacillota), and the genus *RB41* of the phylum Acidobacteria (Fig. 1E). The enriched fungal zOTUs were from the genera *Talaromyces*, *Exophiala*, and *Cladophialophora*, all belonging to the class Eurotiomycetes of the phylum Ascomycota (*P* < .050 by EdgeR & ALDEx2). The 4 reduced fungal zOTUs were affiliated with the phyla Mortierellomycota and Ascomycota (*P* < .050 by EdgeR and ALDEx2). The number of enriched and reduced fungal zOTUs was 13, which was much fewer than that of bacterial zOTUs (131 zOTUs). To test whether it was caused by vast differences in bacterial and fungal community richness (Supplementary Fig. 2A and B), we rarefied the bacterial zOTU table to match the number of fungal zOTUs (10 391 sequences) and repeated the EdgeR and ALDEx2 analysis 10 times. Despite this, the number of enriched and reduced fungal zOTUs remained substantially lower than that of bacterial zOTUs (t = 33.215, *P* < .001 by *t*-test; Fig. 1C and D), suggesting that the large differences in bacterial and fungal community richness have a negligible effect.

### Genomic traits and functional genes of microbial communities

**Figure 2:**
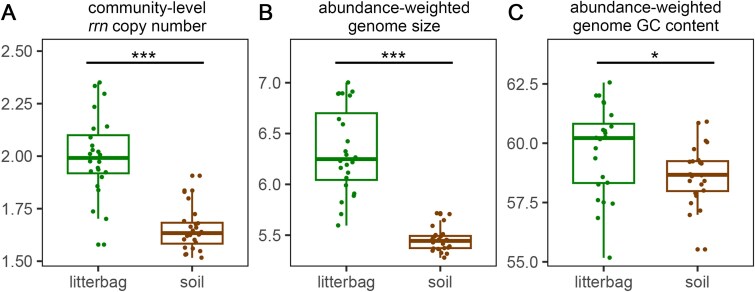
Genome traits of bacterial communities in litterbags and bulk soils. The (A) community-level *rrn* copy number, (B) abundance-weighted genome size (Mbp), and (C) abundance-weighted genome GC content (%) of bacterial communities in litterbags and bulk soils.

The community-level *rrn* copy number of the bacterial communities in litterbags was 2.00 ± 0.19 on average, higher than that in bulk soil (1.65 ± 0.10; F = 62.921, *P* < .001 by LMM; Fig. 2A). Similarly, the abundance-weighted genome size (F = 105.787, *P* < .001 by LMM; Fig. 2B) and genome GC content (F = 5.372, *P* < .050 by LMM; Fig. 2C) of bacterial communities were higher in litterbags than in bulk soil.

Microbial functional compositions, detected by GeoChip 5.0 M [29], a comprehensive and high-throughput functional gene microarray, exhibited differences between litterbags and bulk soils (*P* < .001 by Adonis, Anosim, and MRPP; Supplementary Fig. 3 and Supplementary Table 1). Alike the taxonomic composition of bacterial and fungal communities, the functional gene compositions also exhibited higher dispersion in litterbags than in bulk soil (*P* < .001 by ANOVA; Table 1), possibly arising from random colonization of microbial functional groups in litterbags. However, the correlations between functional richness and taxonomic richness were not significant in both litterbags and bulk soil (Supplementary Fig. 5).

A total of 28 bacterial and 21 fungal genes associated with carbon decomposition were detected, with 3964 probes in total (Supplementary Table 2). Despite high taxonomic diversity (Fig. 1A and B), functional genes involved in cellulose decomposition (e.g. *endoglucanase* and *cellobiase*) were enriched in litterbag samples (*P* < .050 by RR, Supplementary Table 2). Since cellulolytic microbes native to soil can generally utilize a variety of other carbohydrates in addition to cellulose [10], many genes involved in decomposing starch (e.g. *amyA* and *glucoamylase*), hemicellulose (e.g. *ara* and *Mannanase*), chitin (e.g. *acetylglucosaminidase*), glyoxylate cycle (e.g. *aceB*), cutin (e.g. *cutinase*), and lignin (e.g. *mnp*) were also enriched (Supplementary Table 2).

### Warming effects on cellulose mass loss and microbial communities

**Figure 3:**
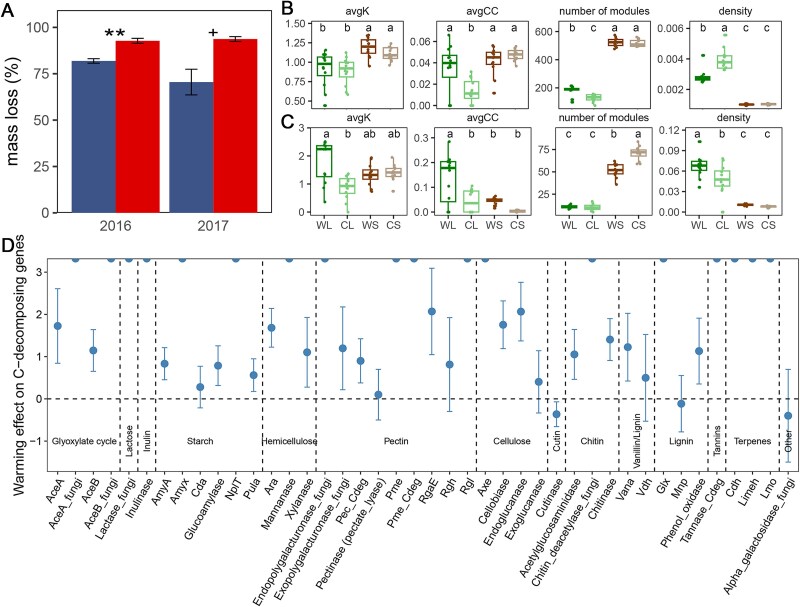
Warming effects on (A) cellulose mass loss, the topological properties of (B) bacterial and (C) fungal networks, and (D) functional genes in litterbags. For each carbon-decomposing gene, the RR was calculated as the summed relative abundances of gene probes in WL samples relative to those in CL samples. Error bars indicate 95% confidence intervals of the RR analysis. The points on the top of the box in panel D represent genes only observed in litterbags. avgK, average degree.

Warming treatment stimulated mass loss of cellulose paper by 11.7% in 2016 (β = 10.841, *P* < .010 by LMM; Fig. 3A). In 2017, it similarly led to a marginally significant increase in cellulose paper mass loss (β = 23.194, *P* = .062 by LMM; Fig. 3A). Compared to control samples, the α-diversities of bacteria, fungi, and functional genes in warmed samples were unchanged in litterbags but reduced in the soil (*P* < .050; Supplementary Fig. 2). In contrast, warming altered overall bacterial and fungal community composition in both bulk soil and litterbags, as determined by three complementary non-parametric multivariate statistical analyses (*P* < .050 by Adonis, Anosim, and MRPP; Supplementary Table 1). Warming increased the relative abundance of bacterial genera *Aridibacter*, *Flavisolibacter*, *Mycobacterium*, and *Massilia*, and fungal genera *Kurtzmanomyces* and *Talaromyces* in litterbags (*P* < .050 by RR, Supplementary Fig. 6). Conversely, warming decreased the relative abundance of bacterial genera *Pedomicrobium*, *Nevskia*, *Dyella*, and *Streptacidiphilus*, and fungal genus *Cladophialophora* in litterbags (*P* < .050 by RR, Supplementary Fig. 6). The interactive effect of warming and year was insignificant (*P* > .050 by Adonis, Supplementary Table 3), suggesting that warming effects on both bacterial and fungal community compositions were not influenced by annual variability.

We generated RMT-based MENs to evaluate potential microbial co-existence patterns (Supplementary Table 4). Consistent with the lower α-diversities observed in litterbags, the network sizes in litterbags were smaller than those in the bulk soil, as indicated by fewer nodes and links (Supplementary Table 4 and Supplementary Fig. 7). In litterbags, warming increased the avgCC (*P* < .010 by LMM) and the number of modules (*P* < .001 by LMM) in bacterial networks, but decreased their density (*P* < .001 by LMM; Fig. 3B). In contrast, warming increased the average degree (*P* < .001 by LMM), average CC (*P* < .010 by LMM), and network density (*P* < .050 by LMM) of fungal networks in litterbags (Fig. 3C). Similar patterns of sub-network properties were observed in all 3 years (Supplementary Fig. 8 and Supplementary Fig. 9).

The community-level *rrn* copy number, abundance-weighted genome size, and abundance-weighted genome GC content were not affected by warming in either the litterbags or bulk soil (*P* > .050 by LMM). In addition, the overall functional composition of both litterbags and bulk soils remained unchanged under the warming treatment (*P* > .050 by Adonis, Anosim, and MRPP; Supplementary Table 1). However, warming enriched functional genes associated with cellulose decomposition in litterbags, including genes *axe*, *cellobiase*, and *endoglucanase* across all 3 years (Fig. 3D). Most genes involved in the decomposition of other carbon compounds, including those related to decomposing glyoxylate cycle (e.g. *aceA* and *aceB*) starch (e.g. *amyA*, *glucoamylase*, *nplT*, and *pulA*), hemicellulose (e.g. *ara*, *mannanase*, and *xylanase*), pectin (e.g. *endopolygalacturonase*, *exopolygalacturonase*, *pme*, and *rgl*), chitin (e.g. *acetylglucosaminidase*, *chitin deacetylase*, and *chitinase*), lignin (e.g. *glx* and *phenol oxidase*), and terpenes (e.g. *cdh* and *limeh*) were also increased in abundance or only detected under warming in litterbags (Fig. 3D).

### Community assembly mechanisms

Annual soil temperature was the most important environmental factor that correlated with bacterial and fungal community compositions in litterbags (*P* < .050 by partial Mantel; Supplementary Table 5). Moisture in the sampling month was correlated with bacterial community compositions (r = 0.180, *P* < .050 by partial Mantel) but not fungal community compositions (r = 0.037, *P* > .050 by partial Mantel; Supplementary Table 5). However, environmental factors explained only a small proportion of the variation in bacterial (10.8%) and fungal (5.6%) communities in litterbags, while biotic factors (i.e., topological properties of networks such as the numbers of nodes and links, average degree, avgCC, average path distance, modularity, and density) explained 22.4% of variations in bacterial communities and 6.0% of variations in fungal communities (MRM analysis, Table 2). Most microbial community variations could not be explained by either abiotic or biotic factors, as the unexplained proportion was 67.9% for bacteria and 88.4% for fungi in litterbags (Table 2).

**Table 2:** Relative contributions of abiotic factors and biotic factors to bacterial and fungal communities based on MRM.

	Litterbag		Soil	
	R^2^	*P*	R^2^	*P*
Bacterial communities				
Abiotic factors	0.109	**0.009** ^**a**^	0.171	**0.001**
Biotic factors	0.212	**0.001**	0.044	**0.012**
Unexplained	0.679	NA	0.785	NA
Fungal communities				
Abiotic factors	0.056	**0.001**	0.070	**0.011**
Biotic factors	0.060	**0.021**	0.245	**0.001**
Unexplained	0.884	NA	0.685	NA

a Significant differences (*P* < .050) are shown in bold.

The tNST values for bacterial communities in both litterbags and bulk soils were greater than 0.50, suggesting that these communities were predominantly assembled by stochastic processes (Supplementary Fig. 10A). Fungal communities also exhibited a tNST value of >0.50 in litterbags, while the tNST value was 0.50 in bulk soil, suggesting more deterministic assembly processes in soil samples (Supplementary Fig. 10B). Functional community assembly in litterbags shifted from more deterministic processes (tNST = 0.45) to more stochastic processes (tNST = 0.58; *P* < .050 by Wilcoxon test) under warming (Supplementary Fig. 10C). However, functional community assembly was more deterministic in bulk soil than in litterbags, exhibiting tNST values of <0.50 in bulk soil (*P* < .001 by Wilcoxon test; Supplementary Fig. 10C).

A positive correlation (r = 0.291–0.605, *P* < .050 by Mantel test) was observed between microbial taxonomic dissimilarity and functional dissimilarity in bulk soils, suggesting that taxonomic and functional composition was coupled (Supplementary Table 6). This correlation was weaker under warming (r = 0.291, *P* < .050 for bacteria; r = 0.292, *P* < .050 for fungi) than under control (r = 0.605, *P* < .001 for bacteria; r = 0.373, *P* < .050 for fungi). In contrast, there was no such correlation between microbial taxonomic dissimilarity and functional dissimilarity in litterbags (Supplementary Table 6).

### Warming effects on soil carbon cycling

Across 3 years of the warming treatment (2016–2018), the average surface soil temperature increased by 1.8°C (β = 1.718, *P* < .001 by LMM) compared to the control plots, while soil moisture decreased by 17.0% (β = −0.619, *P* < .010 by LMM; Supplementary Fig. 11). Warming also significantly increased the total soil respiration (β = 1.075, *P* < .050 by LMM), while other environmental factors remained unchanged (Supplementary Fig. 11).

**Figure 4:**
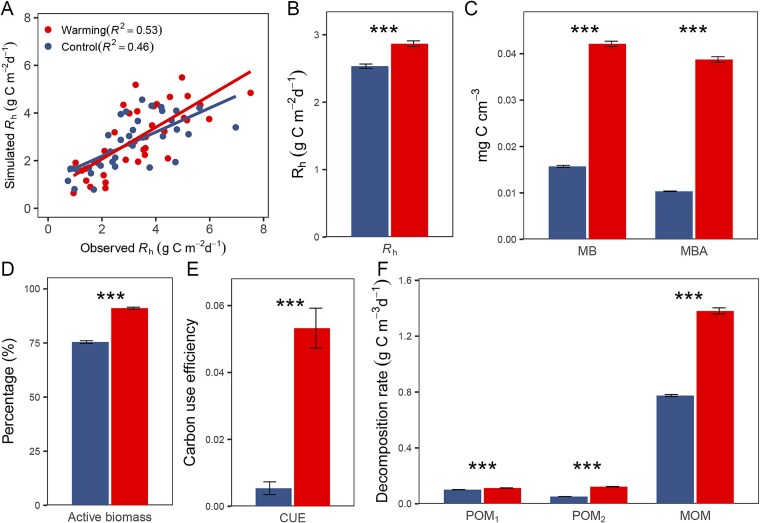
MEND model performance and warming effects on (B) heterotrophic respiration, (C) MB, MBA, (D) percentage of MBA, (E) carbon use efficiency, and (F) decomposition rates of carbon pools based on model simulations. (A) Comparison between gMEND-simulated and observed heterotrophic respiration (*R*_h_) under control and warming conditions. Asterisks indicate significant differences between control model and warming model: ^*^P < .050; ^**^P < .010; ^***^P < .001.

We further estimated the warming effect on soil ecosystems using the MEND model, which takes microbial processes into account [41, 42]. We referred to the MEND model calibrated with only traditional observations, such as heterotrophic respiration (*R*_h_) and MBC, as the traditional MEND model (tMEND). Meanwhile, we designated the MEND model calibrated with supplementary cellulose-decomposing genes as the gene-informed MEND model (gMEND). In addition to the best fit between observed and simulated *R*_h_, we constrained the gMEND model by attaining the highest correlation between gMEND-modeled hydrolytic enzyme concentrations and cellulose-decomposing gene abundances. As a result, the gMEND model was able to effectively simulate the observed soil *R*_h_ under both control (R^2^ = 45.8%) and warming conditions (R^2^ = 53.3%; Fig. 4A). Compared to tMEND model, the parameter uncertainty (coefficient of variation) of gMEND model was reduced for both control and warming models (Supplementary Fig. 12).

Model simulations showed that warming yielded a 13.1% rise in *R*_h_ (t = 6.376, *P* < .001 by *t*-test; Fig. 4B), which was consistent with field observations (Supplementary Fig. 11). Warming increased MB (t = 42.745, *P* < .001 by *t*-test), active MB (MBA; t = 47.308, *P* < .001 by *t*-test; Fig. 4C), microbial active fraction (t = 20.440, *P* < .001 by *t*-test; Fig. 4D) and carbon use efficiency of soil microorganisms (t = 7.635, *P* < .001 by *t*-test; Fig. 4E). Warming also increased the decomposition rates of all three carbon pools in the MEND model (12.0% increase of the decomposition rate of oxidative enzyme-associated particulate organic carbon, 137.4% increase of the decomposition rate of hydrolytic enzymes-associated particulate organic carbon, and 78.2% increase of the decomposition rate of mineral associated organic carbon, *P* < .001 by *t*-test; Fig. 4F).

## Discussion

In this study, we conducted a multi-year in situ litterbag experiment to investigate the effect of warming on the taxonomic and functional compositions of microbial communities involved in cellulose decomposition. Our results showed that the cellulose recruited phylogenetically diverse microorganisms from the bulk soil (Fig. 1), which were enriched in functional genes related to the decomposition of cellulose and other carbon substrates (Fig. 3). In addition, there were no significant correlations between functional richness and taxonomic richness (Supplementary Fig. 5), which was suggestive of high functional redundancy, a phenomenon where multiple species or taxa perform similar ecological roles or functions within a community [55]. Although we found no significant effect of warming on the overall functional composition of the community (Supplementary Table 1), warming increased the relative abundances of most carbon-decomposing genes (Fig. 3). In addition, warming altered the overall taxonomic compositions of microbial communities in litterbags (Supplementary Table 1). This discrepancy between taxonomic and functional responses to warming might be attributed to different assembly mechanisms; deterministic processes dominated functional assembly, while stochastic processes dominated taxonomic assembly (Supplementary Fig. 10). We also incorporated the cellulose-decomposing genes into the MEND model, which improved the model performance and revealed that warming stimulated the decomposition of soil carbon pools (Fig. 4).

## Litterbags versus bulk soil

Understanding the differences between microbial communities in litterbags and bulk soil is essential for identifying the specific recruitment patterns and functional traits of microorganisms involved in cellulose decomposition. We detected 22 138 bacterial zOTUs and 2075 fungal zOTUs in litterbags (Fig. 1 and Supplementary Fig. 2), whose composition was distinct from that in the bulk soil (Supplementary Table 1 and Supplementary Fig. 3). The process of cellulose metabolism in soil environments requires complex interplay between cellulolytic microorganisms and other microbes utilizing the products of cellulose hydrolysis, such as small oligosaccharides and organic acids [10]. This collaborative effort can lead to the detection of numerous taxa in litterbags, many of which may not play a direct role in cellulose decomposition and are thus considered to be “noise”. Nevertheless, we identified several enriched bacterial zOTUs from the phyla Proteobacteria, Bacteroidetes, and Actinobacteria in litterbags (Fig. 1E), consistent with previous studies isolating many cellulolytic bacteria within these phyla [56, 57].

We identified a bacterial zOTU affiliated with the closed-related genera *Burkholderia-Caballeronia-Paraburkholderia* that was enriched in litterbags, showing the highest relative abundance of 10.71% (zOTU1; Fig. 1E). The genus *Burkholderia* accounted for 21.5% of bacterial communities in trophic leaf litter [58] and contributed to carbon accumulation from cellulose in forest litter based on stable isotope probing [59], suggesting its potential importance in plant litter decomposition. The order Rhizobiales had the highest number of enriched bacterial ZOTUs (12/54), in line with previous studies that revealed their high cellulolytic potential [60]. Some members of the Rhizobiales bacteria encode surface attachment proteins and exhibit morphology with filamentous appendages, suggesting their importance in decomposing POM [61]. In contrast to the high number of enriched bacterial taxa, there were far fewer enriched fungal taxa in litterbags (Fig. 1C and D). Many fungal species are capable of cellulose decomposition, while cellulolytic bacteria are concentrated within a few taxa [10]. In addition, fungi are more patchily distributed in soils, making it more difficult to consistently identify enriched fungal taxa across multiple samples compared to bacteria. The enriched fungal taxa identified in our study were clustered in the class Eurotiomycetes of the phylum Ascomycota, known to widely possess the Cellobiohydrolases family GH7 [62].

Microbial functional traits are more directly linked to organism fitness and ecosystem processes than phylogenetic biomarkers [63]. Rapid microbial growth requires a higher capacity to synthesize proteins, which is commonly indicated by the high number of *rrn* copies in the genome [64]. In our study, the community-level *rrn* copy number was higher in litterbags than in bulk soil (Fig. 2A), suggesting that bacterial communities in litterbags had an overall higher maximum growth rate and lower carbon use efficiency [65]. Similarly, our recent study revealed that copiotrophic coastal sediments favored microbial communities with high *rrn* copy numbers, while oligotrophic ocean water favored microbial communities with low *rrn* copy numbers [34]. The abundance-weighted genome size of bacterial community was also higher in litterbags than in bulk soil (Fig. 2B). One possible explanation is that the cellulose present in litterbags provides a favorable niche for copiotrophic microorganisms, which are less likely to undergo genome streamlining as a mechanism of nutrient conservation under nutrient limitation [66]. Furthermore, the larger abundance-weighted genome size and higher abundance-weighted genome GC content observed in litterbags (Fig. 2B and C) implicate a lower carbon use efficiency [20].

## Warming effects on microbial communities

The interactive effects of warming and year on microbial communities and their networks were insignificant (Supplementary Table 3), suggesting that our results were repeatable and not influenced by annual variability. Warming alters microbial taxonomic composition and co-existence patterns in both bulk soils and litterbags (Supplementary Table 1 and Fig. 3). However, there was no significant change in microbial α-diversity in litterbags under warming (Supplementary Fig. 2), differing from results in bulk soil where warming decreased microbial α-diversity [6, 67]. One physiological response of microorganisms to warming is to reduce substrate affinity, leading to a higher substrate demand [68, 69]. Therefore, the generally high substrate supply in litterbags could meet the high substrate demand and sustain microbial growth to maintain α-diversity. The litterbags provide a cellulose-rich microenvironment that selectively recruits a diverse array of microorganisms from the surrounding soil, many of which possess genes encoding cellulolytic enzymes. Microbial taxa exhibit varying temperature sensitivities, reflecting their different metabolic responses to warming [70]. Accordingly, warming affected microbial community composition in both litterbags and bulk soils (Supplementary Table 1 and Supplementary Fig. 6), as observed previously [7]. Evidenced by significant changes in average CC and density of microbial networks (Fig. 3B), warming altered potential microbial co-existence patterns in litterbags, implying possible changes in network stability [38].

Unlike certain narrow functions dominated by a few phylogenetically specific groups [71], cellulose decomposition can be performed concurrently by taxa that are broadly distributed across the microbial tree of life [10, 72]. A high degree of functional redundancy suggests functional stability in biogeochemical cycles [73], which could contribute to functional resistance to warming. This redundancy enables ecosystems to sustain carbon decomposition even when specific taxa are diminished or removed due to changing conditions, maintaining the stability of pathways [1, 74]. By supporting a continuous supply of functional genes involved in cellulose degradation, functional redundancy acts as a buffer against environmental fluctuations, preserving key biogeochemical cycles [31]. Warming did not alter the overall functional composition of microbial communities (Supplementary Table 1), suggesting that warming decoupled taxonomic and functional composition. This decoupling between taxonomic and functional dissimilarity was further supported by the lack of a significant relationship between taxonomic dissimilarity and functional dissimilarity in litterbags (Supplementary Table 6). On the contrary, no such decoupling was observed in bulk soil (Supplementary Table 6), which may be attributed to the lower within-group dispersion in taxonomic and functional compositions in bulk soil (Table 1). Interestingly, we observed an increase in specific functional genes associated with carbon decomposition in response to warming (Fig. 3D). This finding agreed with many previous studies showing that warming enhanced the potential for microbial cellulose decomposition by increasing the relative abundance of microbial carbon-decomposing genes [75].

Functional redundancy does not necessarily imply community neutrality because species with overlapping metabolic potential might be affected by different combinations of biotic and abiotic factors [76]. In our study, the taxonomic composition showed a predominantly stochastic assembly, while the functional assembly was dominated by deterministic processes (Supplementary Fig. 10). During the establishment of the fungal community in beech wood, the assembly history manipulated by pre-colonizing fungal species dictated the subsequent community composition [77]. This phenomenon, known as the priority effect [78], refers to how the order and timing of species’ arrival influence community development because early-arriving species can alter environmental conditions, such as producing secondary metabolites, which modify the niches available to late-arriving species [79]. In our study, taxonomically distinct cellulolytic species may have been recruited into the litterbags first and altered the trajectory of community assembly, resulting in a high level of stochasticity in microbial taxonomic assembly.

## Warming effects on carbon cycling

The effects of warming on microbial communities have direct consequences for carbon cycling, particularly in SOM decomposition. We have recently demonstrated that developing microbial-explicit ecosystem models with functional traits will empower us to mechanistically simulate extensive-scale experiments, which would otherwise incur substantial costs if implemented in real-world settings. This approach enables us not only to verify experimental results but also to predict their future dynamics with precision [80]. Accordingly, we incorporated the cellulose-decomposing genes into the MEND model. Our refined gMEND model exhibited lower uncertainty compared to the tMEND model while still predicting *R*_h_ well. Subsequent simulation showed that warming increased *R*_h_ (Fig. 4B), which was consistent with our observation that warming stimulated total soil respiration by 32.3% and increased *R*_h_ by 12.1% in soil (Supplementary Fig. 11). We found a smaller increase in *R*_h_ under warming (Fig. 4B) compared to an earlier observation at our study site, which induced a 22.0% increase of *R*_h_ between 2010 and 2012 [81]. Long-term warming might foster thermal adaptation within microbial communities, resulting in a smaller increase in *R*_h_ in later years. Accordingly, we found that the relative abundances of carbon-decomposing genes in the warming samples increased during the initial 5 years but decreased to similar levels to the control samples by the 6th and 7th years [31]. Furthermore, our model simulations showed that warming stimulated soil microbial activity and carbon-decomposing rates, especially for stable mineral-associated organic carbon (Fig. 4), which was consistent with previous study that MOM was more sensitive to warming than POM in grasslands [82]. In addition, a recent study suggested that rapid shifts in microbial interactions could substantially change the temperature dependence of microbial community respiration [83]. We also observed that warming altered microbial co-existence networks (Fig. 3B and C), which could suggest changes in putative biotic interactions. Future work to incorporate microbial interactions into our gMEND model holds promise for enhancing the model performance.

## Conclusive remarks

In this study, we examined microbial taxonomic and functional communities in litterbags and bulk soils, as well as how they were affected by global warming. Although there was considerable overlap in the microbial populations found in litterbags and bulk soils, their community compositions showed significant differences, with greater dispersion and heterogeneity observed in litterbags. In addition, we provide evidence of functional redundancy in the microbial communities responsible for cellulose decomposition.

Our study revealed significant effects of climate warming on microbial communities in both litterbags and bulk soils, with changes in certain bacterial and fungal genera and increases in genes associated with carbon decomposition. Moreover, our analysis of community assembly mechanisms showed that while abiotic and biotic factors contributed to shaping the composition of microbial communities, most variations in the microbial communities could not be accounted for, suggesting that stochastic processes played a more substantial role in community assembly. Overall, our study highlights the importance of comprehending the composition and assembly dynamics of microbial communities in soil and litterbags, corroborating the recent findings that microbial decomposers exert a broad control on litter decomposition rates [74]. As a result, explicitly incorporating cellulose decomposers in biogeochemical models could provide valuable insights for accurately predicting future carbon dynamics in the face of global warming.

## Supplementary Material

Supp_litterbag_ycae152

## Data Availability

DNA sequences of 16S rRNA gene and ITS amplicons for bulk soil samples are publicly available in NCBI Sequence Read Archive under the accession number PRJNA331185. DNA sequences of 16S rRNA gene and ITS amplicons for litterbag samples are publicly available in NCBI Sequence Read Archive under the accession number PRJNA995218. GeoChip data are publicly available online (www.ncbi.nlm.nih.gov/geo/) with the accession number GSE237659.

## Code availability

The R codes that support the findings of this study are publicly available at https://github.com/sssyf/litterbag.
