# Supplementary material for: Warming stimulates cellulose decomposition by recruiting phylogenetically diverse but functionally similar microorganisms
Source: ISME Commun. 2025 Mar 3;5(1):ycae152. doi: 10.1093/ismeco/ycae152 (PMC11892950; doi:10.1093/ismeco/ycae152)
Supplement: Supp_litterbag_ycae152 [file supp_litterbag_ycae152.docx]

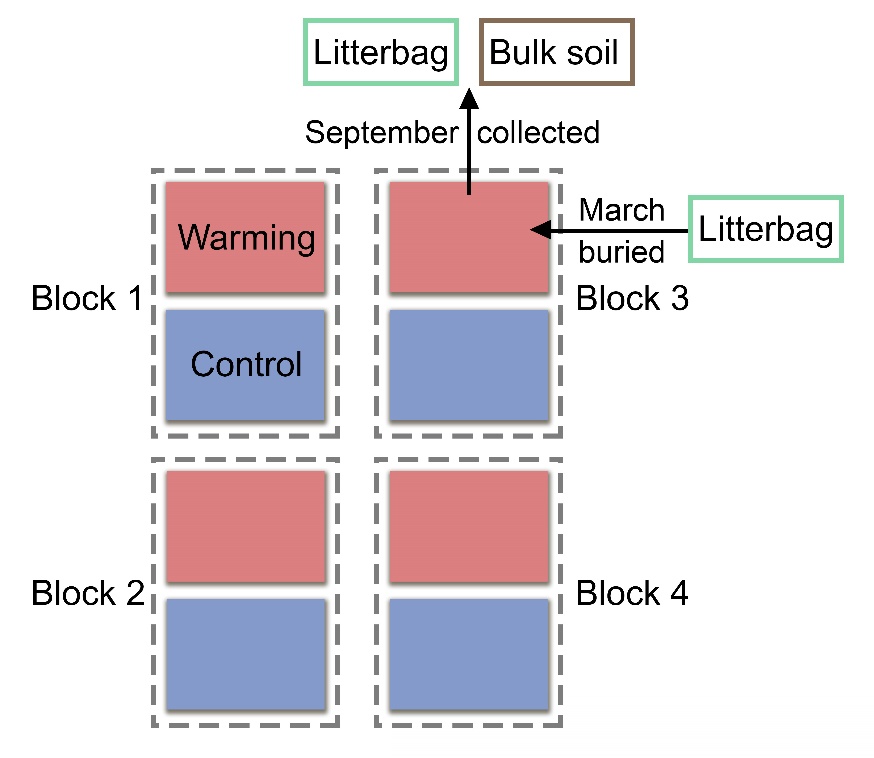


**Fig. S1. Schematic description of field and litterbag experiment.**


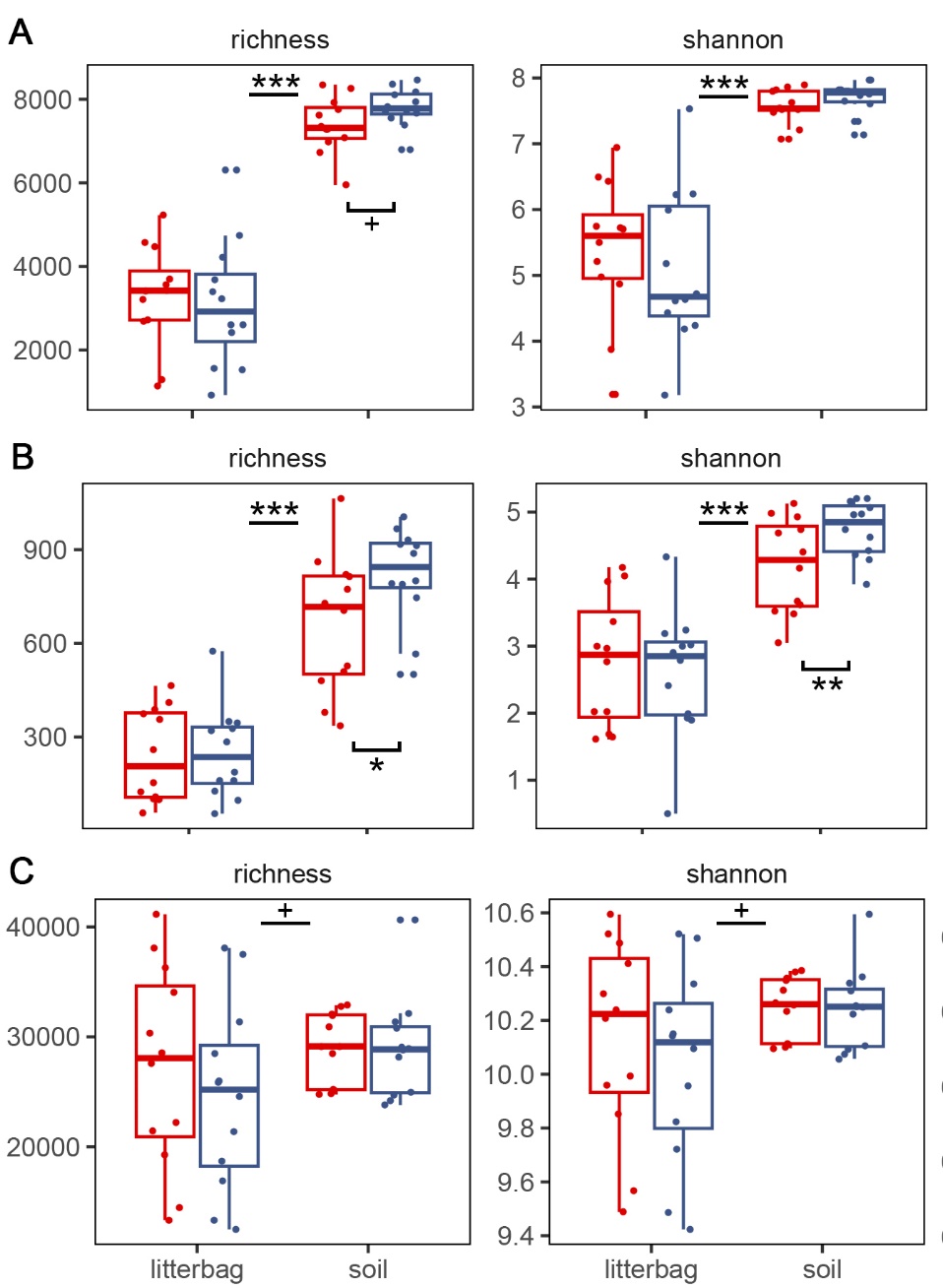


**Fig. S2. The α-diversities of (A) bacterial communities, (B) fungal communities, and (C) functional genes in litterbag and bulk soil samples.** The nodes and boxes colored in red represent warmed samples, and those colored in blue represent control samples. Asterisks indicate significant differences between warmed samples and control samples: ^+^*P* < 0.100; **P* < 0.050; ***P* < 0.010; ****P* < 0.001.


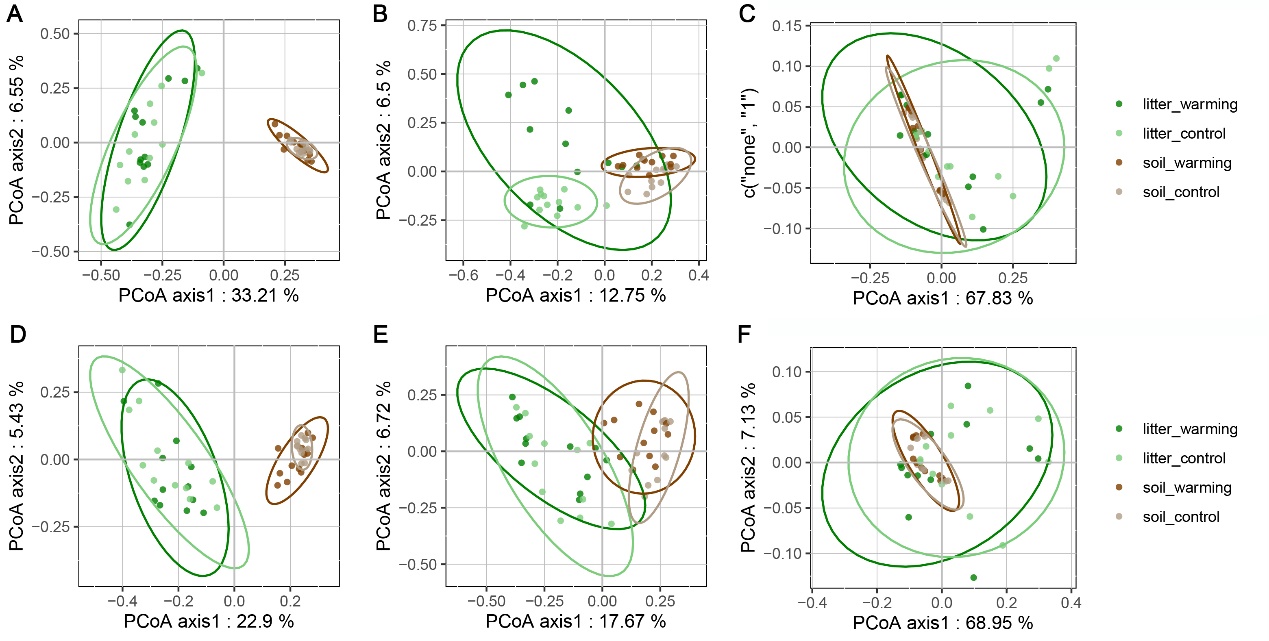


**Fig. S3. Principal Coordinates Analyses of the taxonomic and functional compositions of microbial communities in warmed and control plots.** (**A**) bacterial, (**B**) fungal, and (**C**) functional gene compositions based on Bray-Curtis distance; (**D**) bacterial, (**E**) fungal, and (**F**) functional gene compositions based on Sørenson distance.


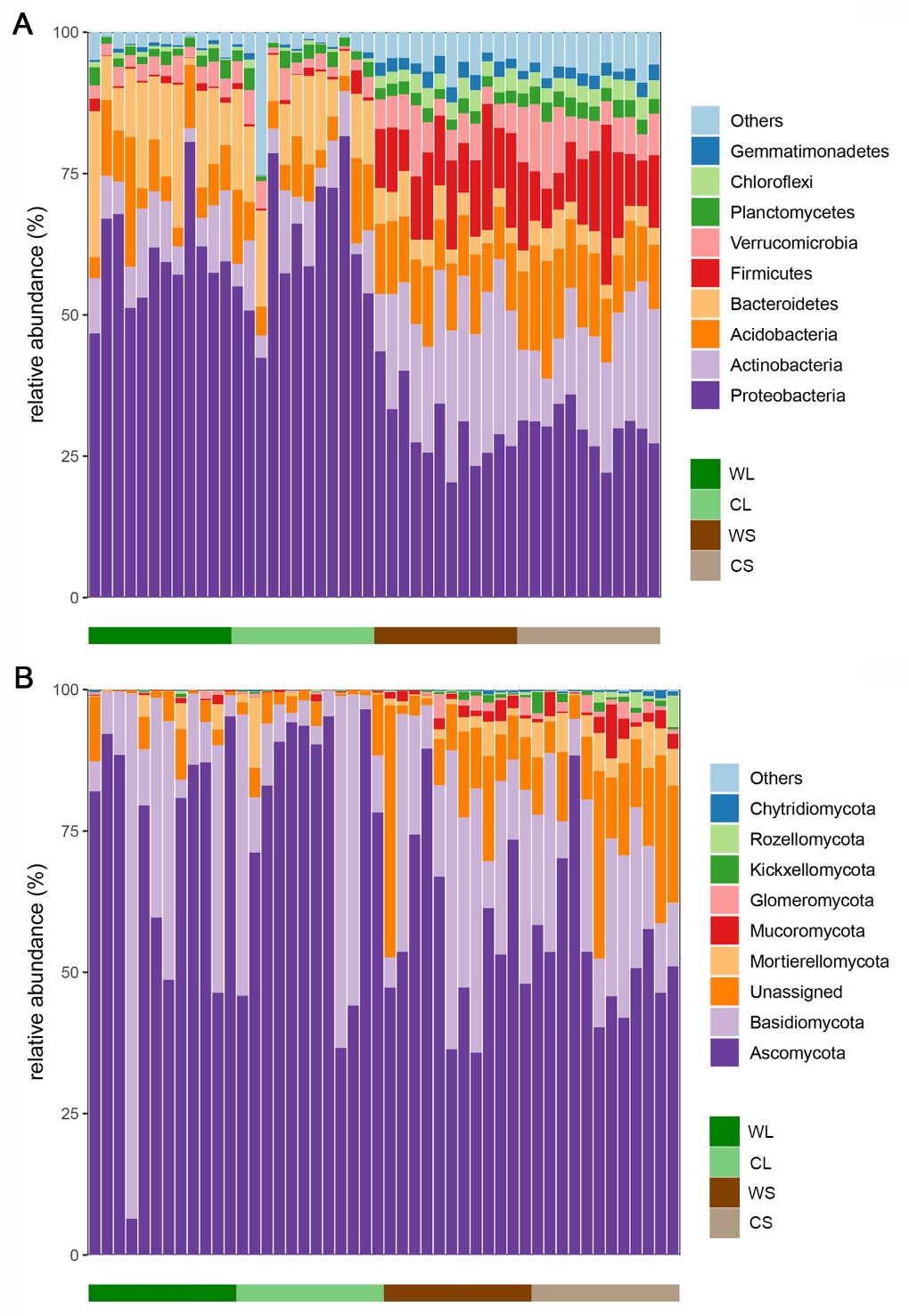


**Fig. S4. Phylum-level compositions of (A) bacteria and (B) fungi.** WL, warmed litterbag samples; CL, control litterbag samples; WS, warmed soil samples; CS, control soil samples.

**
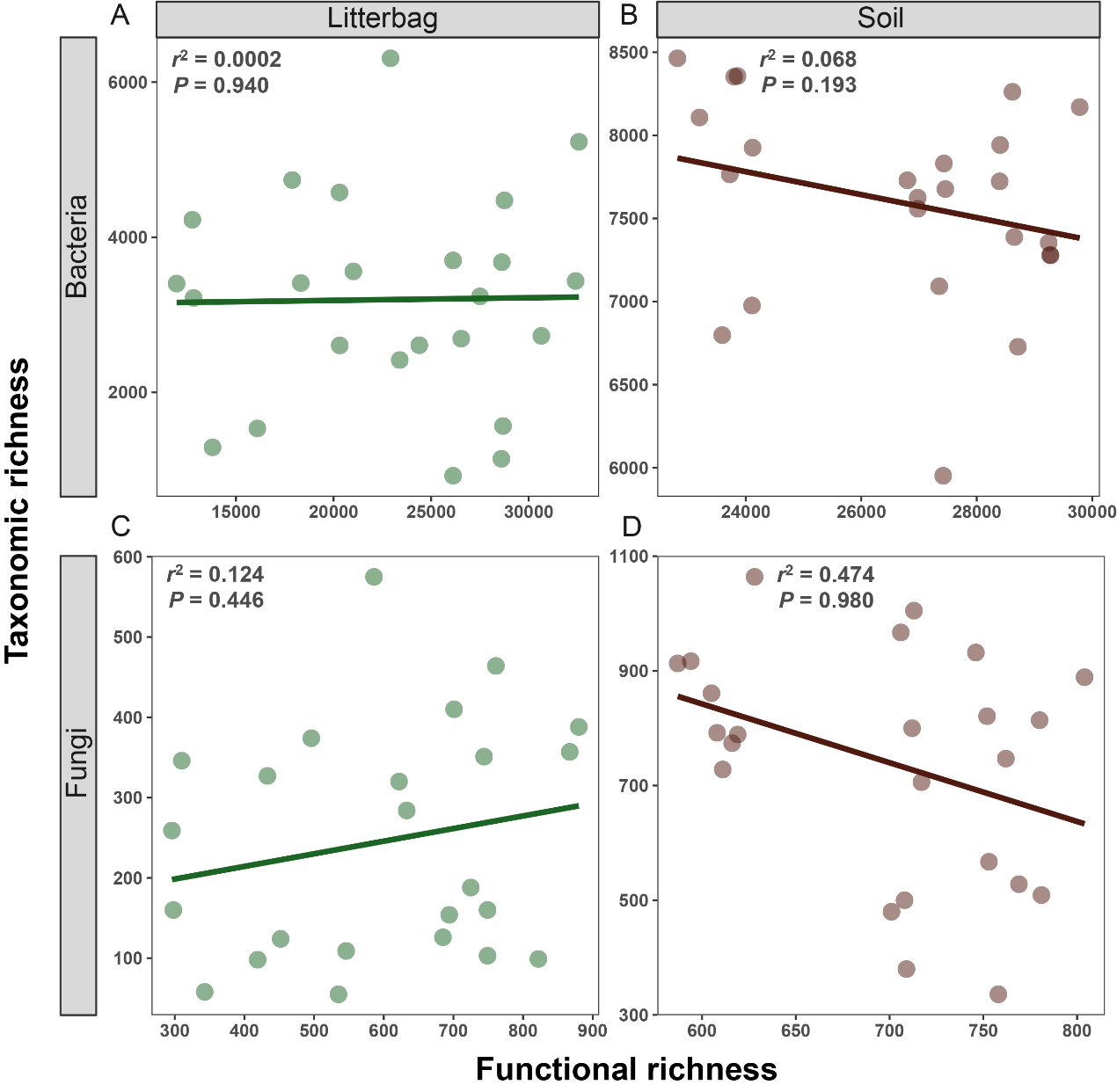
**

**Fig. S5. Relationships between functional and taxonomic richness in litter bags and bulk soil for bacteria and fungi.** The green points represent litterbag samples, while the brown points represent bulk soil samples.


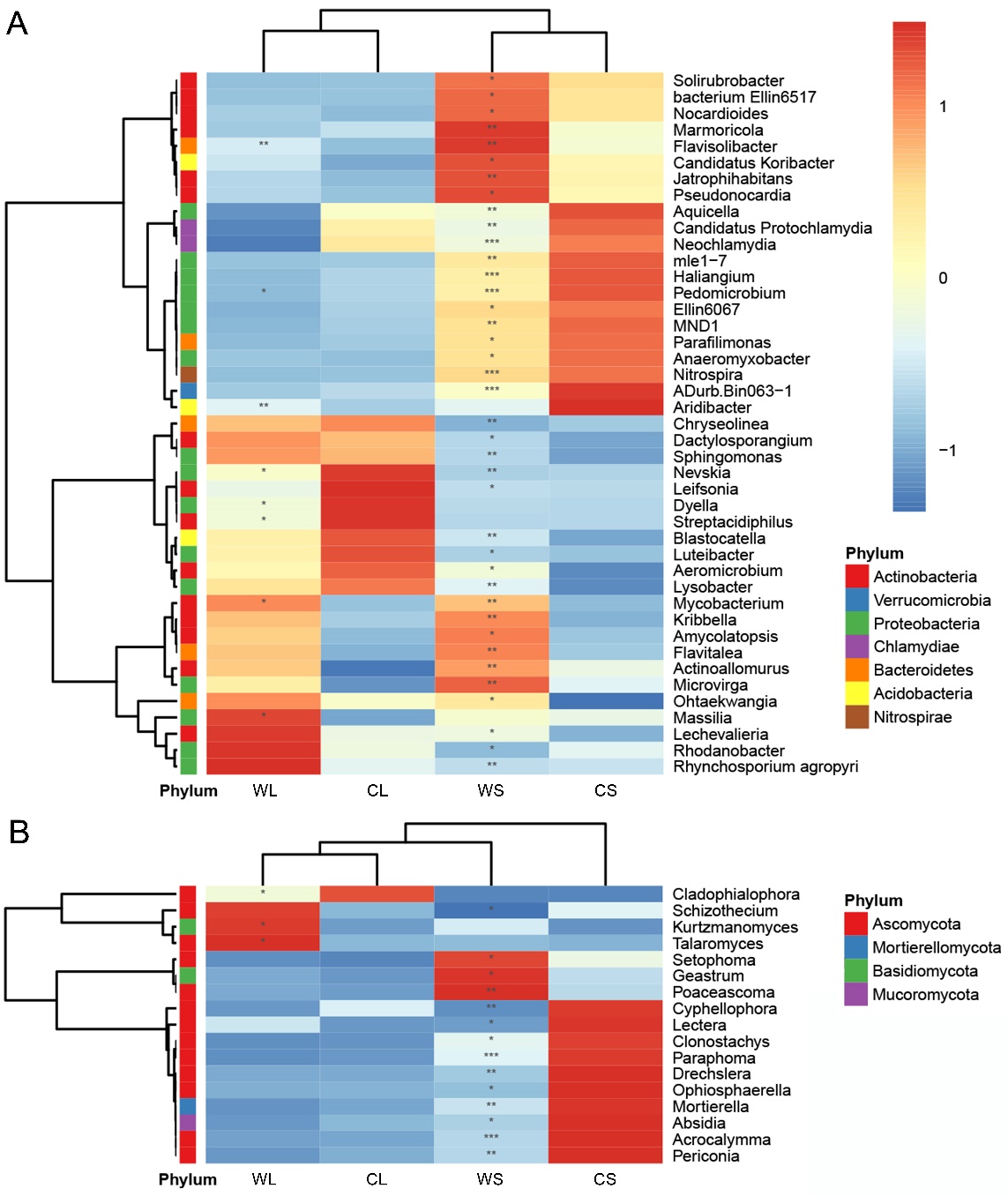


**Fig. S6.** **Heatmaps and clusters of (A) bacterial and (B) fungal genera.** Only genera that had relative abundance > 0.1% and with a significant difference between warmed/control litterbag or warmed/control bulk soil by the linear mixed model are included. WL, warmed litterbag samples; CL, control litterbag samples; WS, warmed soil samples; CS, control soil samples. Asterisks indicate significant differences between warmed and control samples: **P* < 0.050; ***P* < 0.010; ****P* < 0.001.


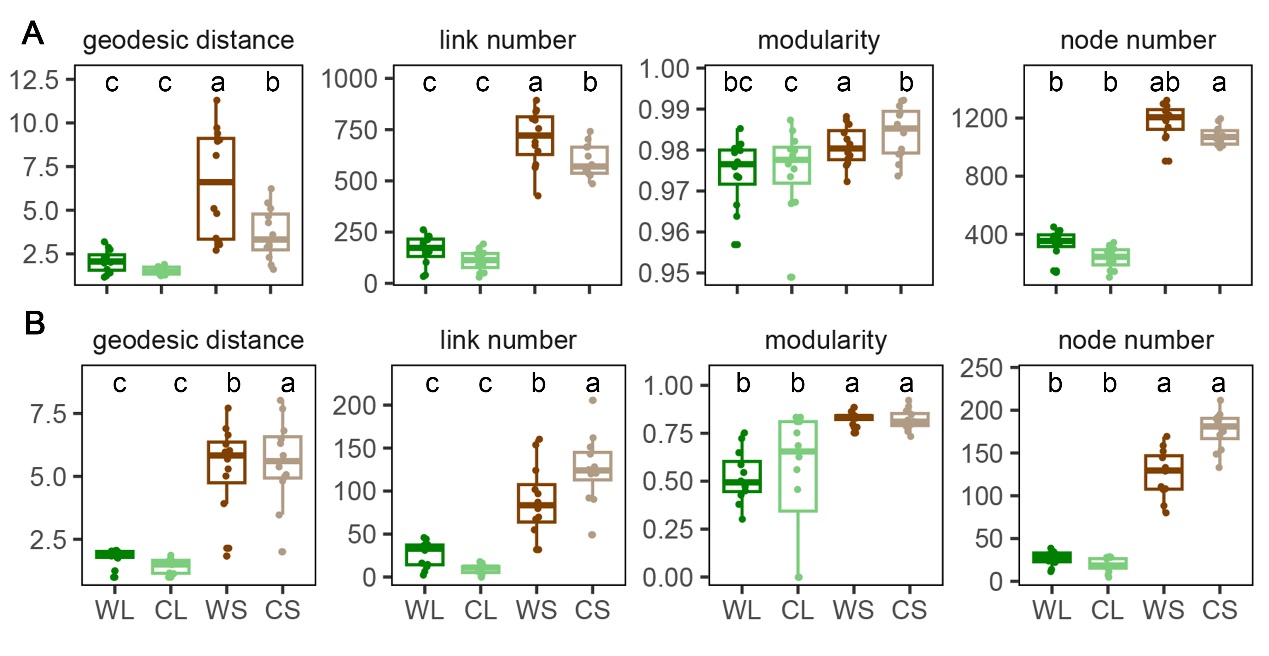


**Fig. S7.** **Other topological properties of (A) bacterial and (B) fungal networks.** WL, warmed litterbag samples; CL, control litterbag samples; WS, warmed soil samples; CS, control soil samples.


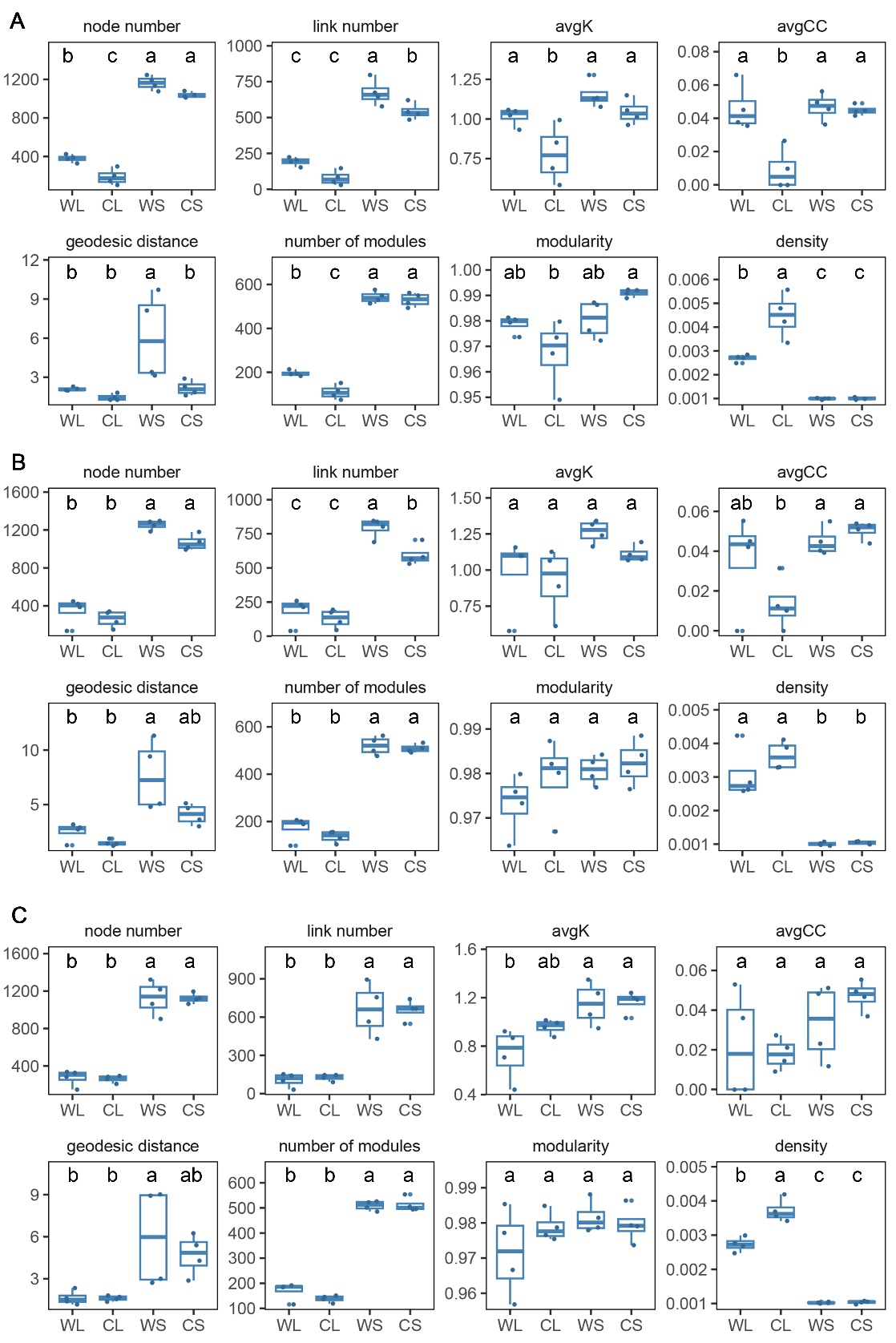


**Fig. S8. Topological properties of bacterial networks in (A) 2016, (B) 2017, and (C) 2018.** WL, warmed litterbag samples; CL, control litterbag samples; WS, warmed soil samples; CS, control soil samples.


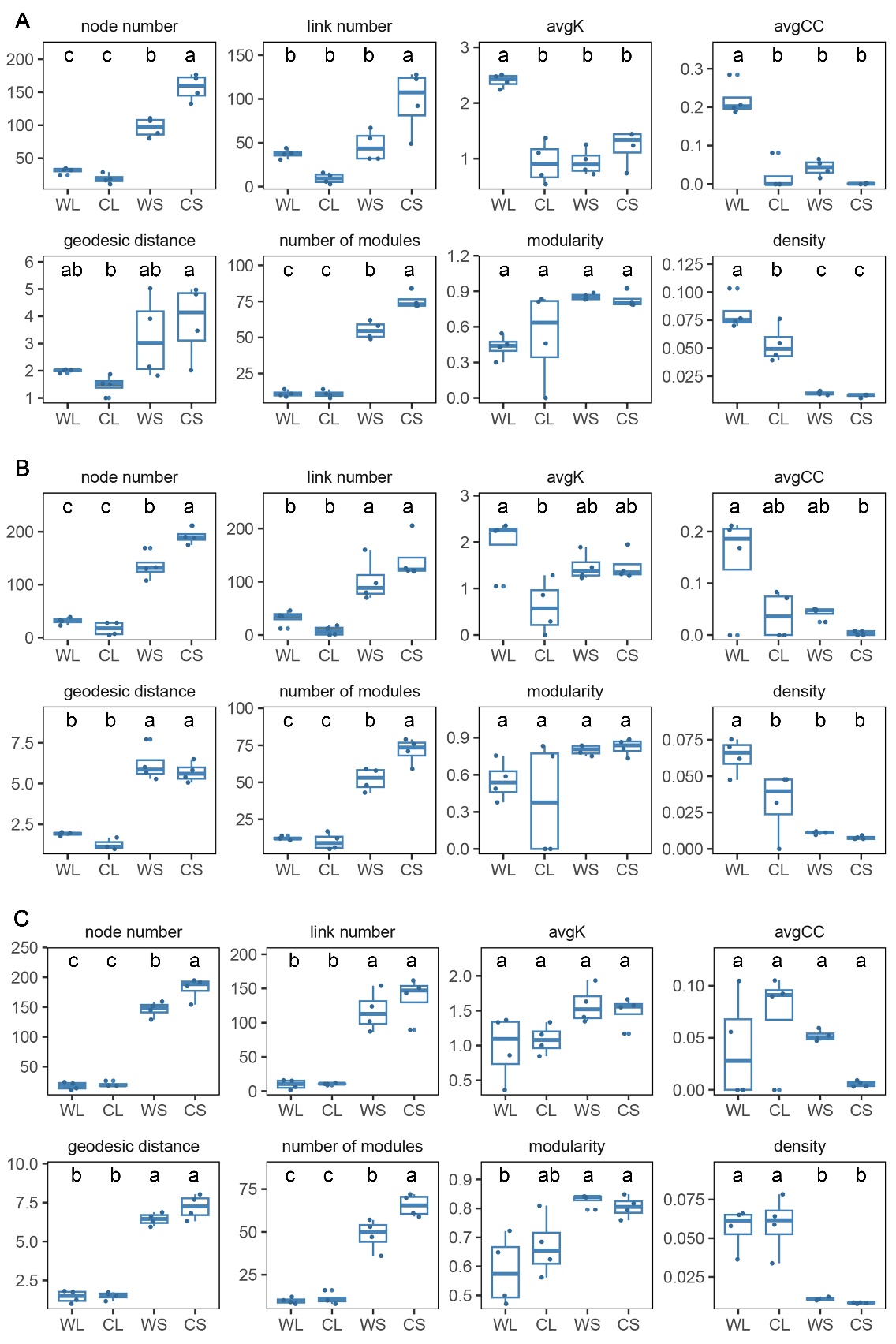


**Fig. S9. Topological properties of fungal networks in (A) 2016, (B) 2017, and (C) 2018.** WL, warmed litterbag samples; CL, control litterbag samples; WS, warmed soil samples; CS, control soil samples.
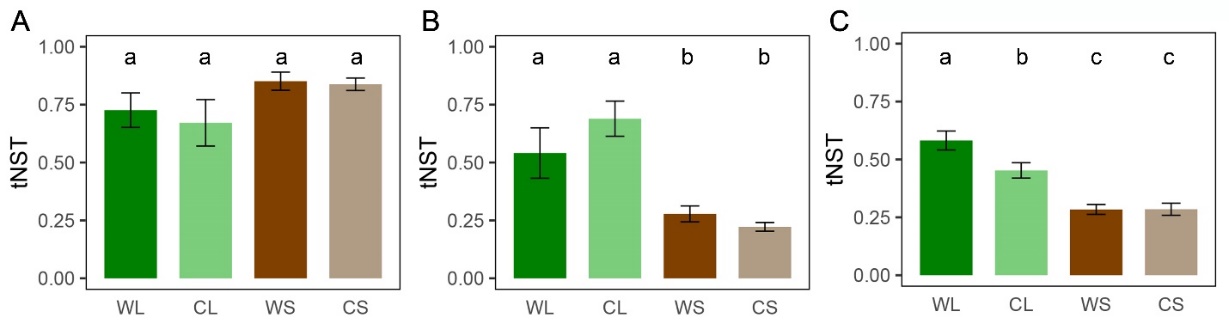


**Fig. S10. Assembly processes of microbial taxonomic and functional communities in litterbags and bulk soils. (A)** bacterial community, **(B)** fungal community, and **(C)** functional genes. WL, warmed litterbag samples; CL, control litterbag samples; WS, warmed soil samples; CS, control soil samples.


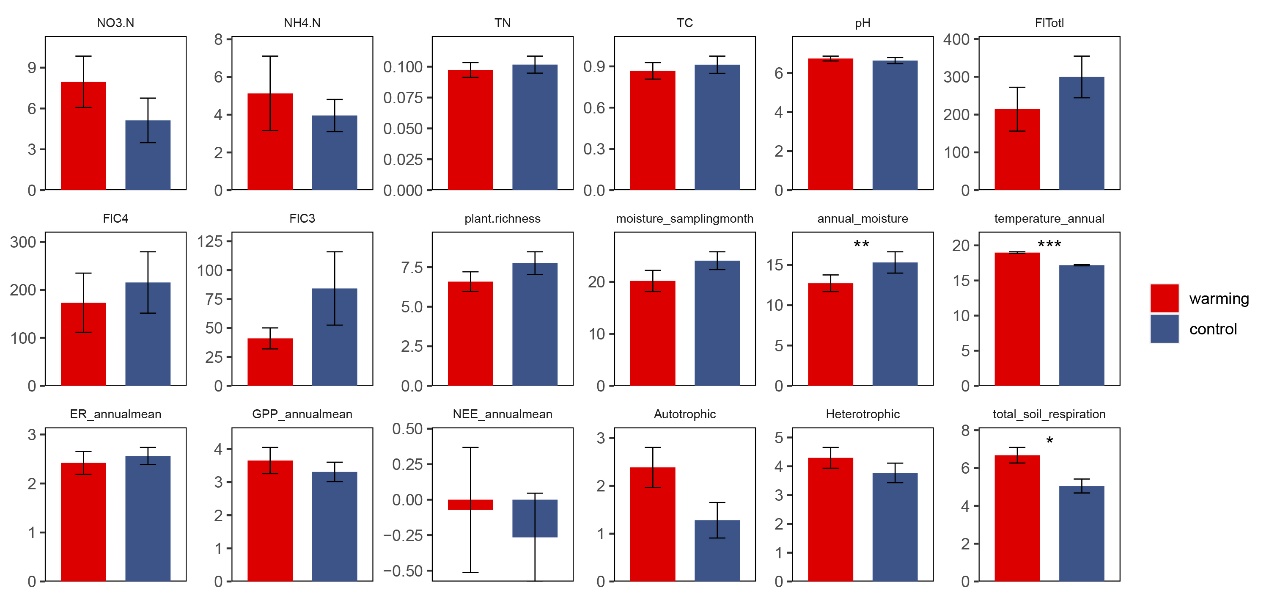


**Fig. S11. Warming effects on environmental factors.** Asterisks indicate significant differences between warmed and control samples: ^+^*P* < 0.100; **P* < 0.050; ***P* < 0.010; ****P* < 0.001.


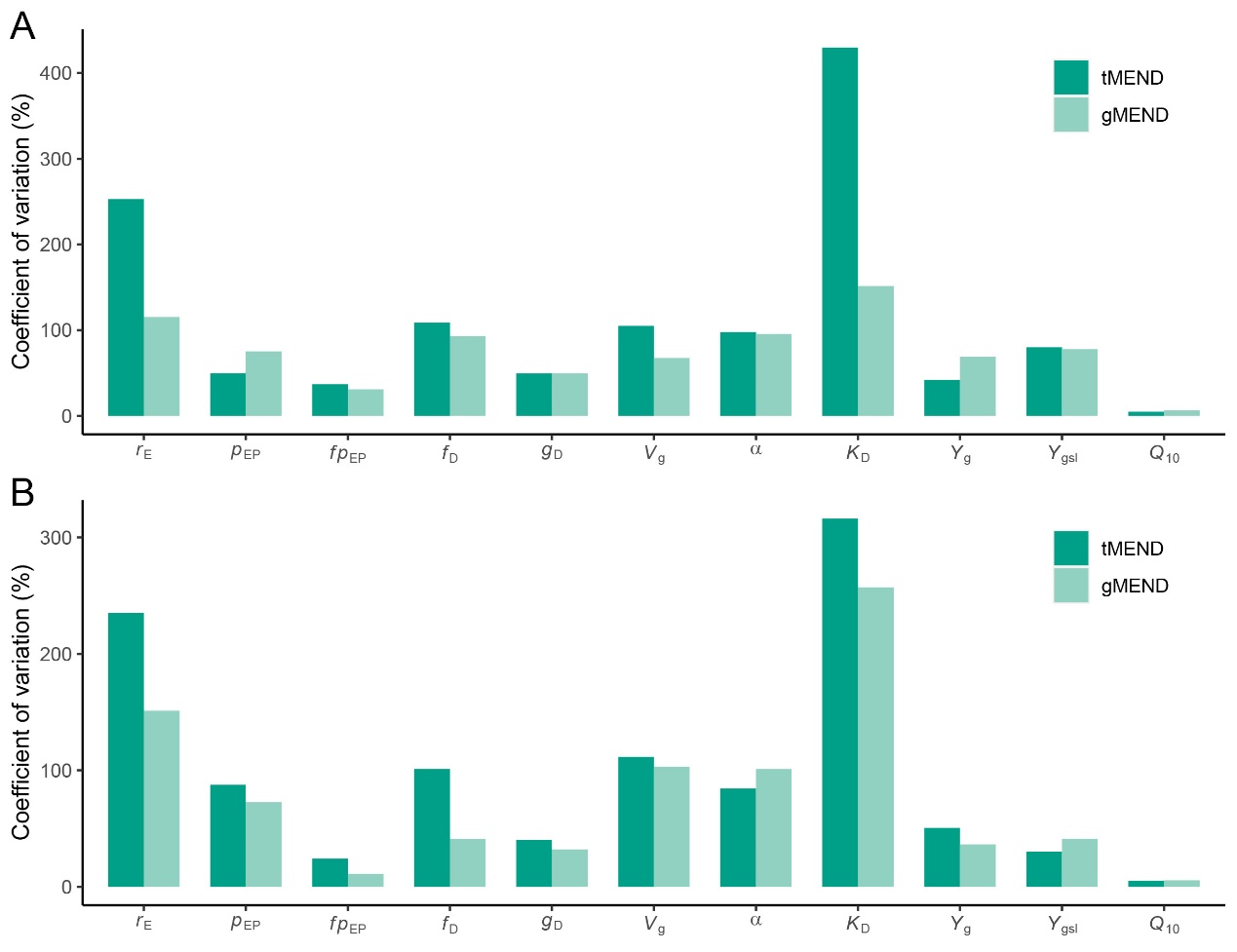


**Fig. S12. The parameter uncertainty of the MEND model calibrated with the (A) control samples and (B) warmed samples.** The 11 model parameters are *r*_E_: enzyme turnover rate; *p*_EP_ and *f*_pEM_: two coefficients controlling EP and EM enzyme production rates; *f*_D_: Fraction of decomposed particulate organic matter (POM) allocated to dissolved organic matter (DOM); *g*_D_: fraction of dead microbe allocating to DOM pool; *V*_g_: maximum specific uptake rate of DOM for microbial growth; α: a coefficient relating specific microbial maintenance rate (*V*_mt_) to growth rate (*alpha*= *V*_mt_ / (*V*_g_ + *V*_mt_)); *K*_D_: half-saturation constant for microbial uptake of DOM; *Y*_g_: true growth yield; *Y*_gsl_: Slope for *Y*_g_ dependence of temperature; *Q*_10_: *Q*_10_ for temperature response function, temperature sensitivity of enzyme-catalyzed soil organic matter decomposition.

**Table S1. Differences in compositions of the bacterial, fungal, and functional gene communities.**

| groups |  | Adonis | | Anosim | | MRPP | |
| --- | --- | --- | --- | --- | --- | --- | --- |
|  | distance | statistic F | *p* | statistic R | *p* | statistic δ | *p* |
| bacterial community | | | | | | | |
| L versus S^a^ | Bray-Curtis | 21.469 | **0.001^b^** | 0.950 | **0.001** | 0.641 | **0.001** |
|  | Sørenson | 12.398 | **0.001** | 0.842 | **0.001** | 0.643 | **0.001** |
| WL versus CL | Bray-Curtis | 1.494 | **0.034** | 0.109 | **0.023** | 0.721 | 0.061 |
|  | Sørenson | 1.463 | **0.018** | 0.125 | **0.023** | 0.686 | **0.013** |
| WS versus CS | Bray-Curtis | 2.533 | **0.001** | 0.297 | **0.001** | 0.537 | **0.001** |
|  | Sørenson | 2.272 | **0.001** | 0.518 | **0.001** | 0.578 | **0.001** |
| fungal community | | | | | | | |
| L versus S | Bray-Curtis | 5.371 | **0.001** | 0.577 | **0.001** | 0.853 | **0.001** |
|  | Sørenson | 7.735 | **0.001** | 0.659 | **0.001** | 0.728 | **0.001** |
| WL versus CL | Bray-Curtis | 2.159 | **0.001** | 0.259 | **0.001** | 0.854 | **0.001** |
|  | Sørenson | 1.629 | **0.020** | 0.154 | **0.013** | 0.750 | **0.019** |
| WS versus CS | Bray-Curtis | 1.928 | **0.002** | 0.226 | **0.001** | 0.811 | **0.001** |
|  | Sørenson | 2.279 | **0.001** | 0.354 | **0.001** | 0.677 | **0.001** |
| functional genes | | | | | | | |
| L versus S | Bray-Curtis | 7.974 | **0.001** | 0.249 | **0.001** | 0.190 | **0.001** |
|  | Sørenson | 8.967 | **0.001** | 0.299 | **0.001** | 0.147 | **0.001** |
| WL versus CL | Bray-Curtis | 0.983 | 0.345 | 0.004 | 0.356 | 0.272 | 0.306 |
|  | Sørenson | 1.299 | 0.247 | 0.031 | 0.175 | 0.209 | 0.185 |
| WS versus CS | Bray-Curtis | 0.856 | 0.385 | 0.003 | 0.327 | 0.108 | 0.386 |
|  | Sørenson | 1.348 | 0.236 | 0.037 | 0.212 | 0.081 | 0.180 |

^a^Abbreviations: L, litterbag samples; S: soil samples; WL, warmed litterbag samples; CL, control litterbag samples; WS, warmed soil samples; CS, control soil samples.

^b^Significant differences (*P* < 0.050) are shown in bold.

**Table S2. Number of carbon degradation gene probes with warming-altered abundances in each gene family in litterbag samples.**

| gene | subcategory | Probe numbers | Litterbag^a^ | Soil^b^ | +^c^ | -^d^ |
| --- | --- | --- | --- | --- | --- | --- |
| bacterial genes | | | | | | |
| AceA | Glyoxylate cycle | 116 | 114 | 110 | 34 | 30 |
| AceB |  | 195 | 188 | 182 | 68 | 43 |
| AmyA | Starch | 1162 | 1100 | 1050 | 407 | 274 |
| AmyX |  | 1 | 1 | 0 | 1 | 0 |
| Apu |  | 1 | 1 | 1 | 0 | 0 |
| Cda |  | 93 | 88 | 84 | 26 | 30 |
| Isopullulanase |  | 3 | 3 | 3 | 1 | 0 |
| NplT |  | 28 | 26 | 23 | 12 | 4 |
| PulA |  | 63 | 58 | 54 | 21 | 16 |
| Ara | Hemicellulose | 172 | 164 | 154 | 58 | 33 |
| Mannanase |  | 69 | 67 | 63 | 24 | 15 |
| Xylanase |  | 157 | 146 | 141 | 47 | 43 |
| Pectinase (pectate_lyase) | Pectin | 51 | 47 | 45 | 22 | 14 |
| Pme |  | 56 | 51 | 51 | 21 | 15 |
| RgaE |  | 70 | 68 | 66 | 27 | 18 |
| Rgh |  | 19 | 19 | 15 | 7 | 2 |
| Rgl |  | 61 | 57 | 57 | 14 | 15 |
| Endoglucanase | Cellulose | 106 | 101 | 91 | 47 | 22 |
| Acetylglucosaminidase | Chitin | 200 | 190 | 177 | 70 | 34 |
| Chitinase |  | 395 | 380 | 354 | 125 | 98 |
| VanA | Vanillin/Lignin | 80 | 75 | 73 | 33 | 13 |
| Vdh |  | 26 | 25 | 24 | 7 | 8 |
| Glx | Lignin | 33 | 33 | 28 | 13 | 10 |
| Mnp |  | 22 | 22 | 19 | 12 | 0 |
| Phenol_oxidase |  | 131 | 124 | 118 | 45 | 31 |
| Cdh | Terpenes | 25 | 24 | 24 | 6 | 8 |
| Limeh |  | 33 | 32 | 33 | 9 | 9 |
| Lmo |  | 4 | 4 | 4 | 1 | 2 |
| Fungal genes | | | | | | |
| AceA_fungi | Glyoxylate cycle | 7 | 7 | 6 | 3 | 2 |
| AceB_fungi |  | 9 | 9 | 9 | 2 | 1 |
| Lactase_fungi | Lactose | 13 | 11 | 11 | 3 | 6 |
| Inulinase | Inulin | 6 | 6 | 6 | 1 | 4 |
| Glucoamylase | Starch | 61 | 57 | 53 | 24 | 10 |
| Endopolygalacturonase_fungi | Pectin | 12 | 12 | 7 | 5 | 2 |
| Exopolygalacturonase_fungi |  | 17 | 16 | 16 | 4 | 6 |
| Pec_Cdeg |  | 34 | 31 | 33 | 8 | 10 |
| Pel_Cdeg |  | 4 | 4 | 4 | 1 | 1 |
| Pme_Cdeg |  | 19 | 17 | 17 | 7 | 4 |
| Axe | Cellulose | 16 | 15 | 14 | 10 | 3 |
| Cellobiase |  | 144 | 140 | 126 | 52 | 34 |
| Exoglucanase |  | 44 | 42 | 39 | 15 | 7 |
| Cutinase | Cutin | 95 | 90 | 92 | 41 | 22 |
| Chitin_deacetylase_fungi | Chitin | 10 | 9 | 8 | 4 | 4 |
| Ligninase | Lignin | 7 | 7 | 7 | 3 | 1 |
| Tannase_Cdeg | Tannins | 19 | 19 | 17 | 7 | 3 |
| Alpha_galactosidase_fungi | Other | 15 | 14 | 11 | 5 | 3 |

^a^Litterbag means the number of gene probes detected in litterbag samples;

^b^soil means the number of gene probes detected in bulk soil samples;

^c^+ denotes abundances of genes significantly increased (response ratio > 0 & *P* > 0.050) in litterbags;

^d^- denotes abundances of genes significantly decreased (response ratio < 0 & *P* > 0.050) in bulk soil. Gene families are sorted and displayed from labile carbon to recalcitrant carbon.

**Table S3. Significance tests of the effects of sample type, warming, and year on microbial community with Permutational multivariate analysis of variance (Adonis).**

|  | bacterial composition | | | | fungal composition | | | | functional composition | | | |
| --- | --- | --- | --- | --- | --- | --- | --- | --- | --- | --- | --- | --- |
|  | Bray-Curtis | | Sørenson | | Bray-Curtis | | Sørenson | | Bray-Curtis | | Sørenson | |
|  | F value | p | F value | p | F value | p | F value | p | F value | p | F value | p |
| sample type | 22.910 | **0.001** | 13.134 | **0.001** | 5.705 | **0.001** | 8.333 | **0.001** | 8.762 | **0.001** | 9.933 | **0.001** |
| warm | 2.314 | **0.043** | 2.461 | **0.012** | 2.400 | **0.002** | 2.743 | **0.001** | 1.214 | 0.237 | 1.604 | 0.143 |
| year | 1.700 | 0.098 | 1.578 | 0.060 | 1.510 | **0.030** | 1.971 | **0.010** | 5.638 | **0.006** | 5.210 | **0.008** |
| sample type x warm | 1.523 | 0.121 | 1.221 | 0.151 | 1.766 | **0.006** | 1.235 | 0.107 | 0.912 | 0.354 | 1.249 | 0.246 |
| sample type x year | 1.482 | 0.118 | 1.286 | 0.129 | 1.475 | **0.023** | 1.523 | **0.038** | 1.881 | 0.11 | 2.140 | 0.108 |
| Warm x year | 1.102 | 0.277 | 1.162 | 0.185 | 0.773 | 0.872 | 1.011 | 0.255 | 0.468 | 0.66 | 0.426 | 0.702 |

**Table S4. Topological properties of the empirical bacterial and fungal networks in each group.**

| groups | S_t_ | R^2^ of power law | nodes | links | avg connectivity | avg path distance | avg cluster coefficient | modularity (no. of modules) |
| --- | --- | --- | --- | --- | --- | --- | --- | --- |
| bacterial network | | | | | | | | |
| WL^a^ | 0.93 | 0.951 | 557 | 400 | 1.436 | 2.985 | 0.050 | 0.983 (177) |
| CL | 0.93 | 0.970 | 395 | 255 | 1.291 | 1.814 | 0.027 | 0.987 (146) |
| WS | 0.93 | 0.826 | 1805 | 1506 | 1.669 | 16.531 | 0.052 | 0.977 (428) |
| CS | 0.93 | 0.974 | 1654 | 1292 | 1.562 | 7.095 | 0.062 | 0.981 (458) |
| fungal network | | | | | | | | |
| WL | 0.85 | 0.683 | 42 | 50 | 2.381 | 2.017 | 0.156 | 0.563 (9) |
| CL | 0.85 | 0.902 | 31 | 26 | 1.677 | 1.883 | 0.054 | 0.662 (9) |
| WS | 0.85 | 0.835 | 198 | 239 | 2.414 | 6.311 | 0.052 | 0.753 (21) |
| CS | 0.85 | 0.861 | 272 | 376 | 2.765 | 5.554 | 0.005 | 0.677 (21) |

^a^Abbreviations: WL, warmed litterbag samples; CL, control litterbag samples; WS, warmed soil samples; CS, control soil samples.

**Table S5. Key environmental factors linked with microbial communities based on partial Mantel test.**

| variables | litterbag | | | | bulk soil | | | |
| --- | --- | --- | --- | --- | --- | --- | --- | --- |
|  | bacteria | | fungi | | bacteria | | fungi | |
|  | r | *P* | r | *P* | R | *P* | r | *P* |
| environmental factors | | | | | | | | |
| pH | -0.072 | 0.770 | -0.083 | 0.796 | 0.317 | **0.003^a^** | 0.196 | **0.039** |
| Total plant biomass | -0.037 | 0.697 | 0.047 | 0.275 | -0.040 | 0.662 | 0.094 | 0.202 |
| C4 plant biomass | 0.122 | 0.103 | -0.099 | 0.836 | -0.087 | 0.758 | 0.059 | 0.341 |
| C3 plant biomass | 0.077 | 0.220 | 0.118 | 0.152 | 0.090 | 0.281 | -0.076 | 0.604 |
| Plant richness | -0.077 | 0.762 | -0.103 | 0.810 | -0.049 | 0.633 | 0.127 | 0.162 |
| Moisture sampling month | 0.180 | **0.026** | 0.037 | 0.317 | 0.036 | 0.381 | -0.122 | 0.872 |
| Annual moisture | 0.027 | 0.368 | -0.027 | 0.584 | 0.094 | 0.198 | 0.155 | 0.089 |
| Temperature annual | 0.188 | **0.008** | 0.228 | **0.002** | 0.257 | **0.001** | 0.174 | **0.009** |
| ecosystem functions | | | | | | | | |
| ER annual mean | -0.013 | 0.543 | 0.070 | 0.217 | 0.141 | 0.100 | -0.018 | 0.572 |
| GPP annual mean | 0.099 | 0.143 | 0.209 | **0.019** | 0.101 | 0.173 | 0.203 | **0.045** |
| NEE annual mean | 0.102 | 0.120 | 0.310 | **0.001** | 0.291 | **0.001** | 0.358 | **0.002** |
| Autotrophic | 0.082 | 0.175 | 0.173 | **0.040** | -0.028 | 0.581 | -0.112 | 0.879 |
| Heterotrophic | 0.057 | 0.227 | 0.022 | 0.371 | 0.132 | 0.083 | 0.044 | 0.303 |
| Total soil respiration | 0.116 | 0.074 | 0.051 | 0.255 | 0.086 | 0.196 | -0.061 | 0.719 |
| cellulose mass loss | | | | | | | | |
| Cellulose mass loss | 0.258 | **0.042** | 0.333 | **0.026** | 0.036 | 0.395 | -0.082 | 0.678 |

^a^Significant differences (*P* < 0.050) are shown in bold.

**Table S6. Correlation between the taxonomic and functional compositions of microbial communities based on the Mantel test.**

| group | statistic r | *P* |
| --- | --- | --- |
| bacterial community composition - functional genes | | |
| litterbag | -0.015 | 0.543 |
| litterbag warming | -0.101 | 0.713 |
| litterbag control | -0.114 | 0.770 |
| soil | 0.405 | **< 0.001** |
| soil warming | 0.291 | **0.015** |
| soil control | 0.605 | **0.000** |
| fungal community composition - functional genes | | |
| litterbag | 0.095 | 0.171 |
| litterbag warming | -0.052 | 0.584 |
| litterbag control | -0.040 | 0.576 |
| soil | 0.356 | **< 0.001** |
| soil warming | 0.292 | **0.012** |
| soil control | 0.373 | **0.029** |

^a^Significant differences (*P* < 0.050) are shown in bold.
